# Cardioneuroablation for Reflex Syncope and Functional Bradyarrhythmias: A State-of-the-Art Review

**DOI:** 10.3390/jcm15156012

**Published:** 2026-08-02

**Authors:** Rodolfo San Antonio, Juan Ismael Almonte, Carlos García-Filloy, Jordi Mercé, Julián Rodríguez-García, Jesús Rodríguez-Silva, Marcos Rodríguez-García, Alfredo Chauca, Andrea Di Marco, Paolo D. Dallaglio, Ignasi Anguera

**Affiliations:** 1 Cardiology Department, Bellvitge University Hospital, L’Hospitalet de Llobregat, 08907 Barcelona, Spain; juanismaelalmonte@gmail.com (J.I.A.);; 2 BIOHEART Group, Cardiovascular, Respiratory and Systemic Disease and Cellular Aging Program, Institut d’Investigació Biomèdica de Bellvitge (IDIBELL), L’Hospitalet de Llobregat, 08907 Barcelona, Spain

**Keywords:** cardioneuroablation, ganglionated plexus ablation, vasovagal syncope, reflex syncope, functional atrioventricular block, sinus node dysfunction, cardiac autonomic nervous system

## Abstract

Cardioneuroablation (CNA), defined as endocardial catheter ablation of atrial ganglionated plexi within the intrinsic cardiac autonomic nervous system, has emerged as a mechanism-directed therapy for cardioinhibitory reflex syncope and functional bradyarrhythmias. By attenuating postganglionic parasympathetic input to the sinoatrial and atrioventricular nodes, CNA targets the autonomic substrate of cardioinhibition rather than merely treating its bradycardic consequence. Over the past two decades, the evidence base has progressed from pioneering single-center experiences to multicenter registries, systematic reviews, meta-analyses, and randomized clinical trials, with reported freedom from recurrent syncope of approximately 80–94% in carefully selected patients at medium-term follow-up. This state-of-the-art narrative review summarizes the rationale, anatomy, patient-selection principles, mapping and ablation strategies, procedural endpoints, clinical outcomes, safety profile, and emerging applications of CNA. Particular emphasis is placed on the unresolved questions that will define the future of the field: the absence, to date, of adequately powered sham-controlled efficacy data; the lack of a universally validated intraprocedural endpoint; the biological and clinical significance of vagal reinnervation; and the gap between expert-center and broader real-world outcomes. Current evidence supports CNA as a promising, anatomically grounded neuromodulatory therapy, but its transition from expert-center innovation to guideline-endorsed treatment will depend on rigorous patient selection, standardized procedural endpoints, and the results of ongoing randomized trials.

## 1. Introduction

Reflex, or neurally mediated, syncope is the leading cause of transient loss of consciousness in otherwise healthy individuals, affecting up to 40% of the general population during life [1]. Although usually benign in terms of mortality, a clinically important subset—particularly patients with a dominant cardioinhibitory phenotype—experiences recurrent, unpredictable, and sometimes traumatic episodes that markedly impair quality of life and restrict professional, social, and recreational activity [2]. In this phenotype, an abrupt increase in parasympathetic outflow, often accompanied by sympathetic withdrawal, produces profound sinus bradycardia, sinus arrest, or paroxysmal atrioventricular (AV) block, resulting in cerebral hypoperfusion [3]. The same autonomic mechanism underlies many cases of functional, vagally mediated sinus node dysfunction and high-grade AV block, particularly in young, athletic patients without structural heart disease [4].

Current therapeutic options leave a substantial gap. Education, trigger avoidance, volume expansion, counterpressure maneuvers, and pharmacological treatment may reduce episode burden, but efficacy is inconsistent in severely symptomatic patients and long-term adherence is often limited. Dual-chamber pacing can prevent asystolic pauses, but it does not modify the autonomic reflex, is ineffective against the vasodepressor component, and exposes young patients with normal hearts to cumulative device-, lead-, and infection-related risks over decades—reasons why guideline-supported pacing is generally restricted to older patients with documented spontaneous asystole [5]. A therapy addressing the autonomic substrate of cardioinhibition, rather than only its electrical consequence, is therefore particularly attractive in younger, device-averse, or highly active patients.

Cardioneuroablation (CNA) was introduced by Pachon and colleagues in 2005 as an endocardial radiofrequency strategy for neurally mediated syncope, functional AV block, and sinus node dysfunction [6]. Its rationale is anatomically specific: postganglionic parasympathetic neurons reside within or immediately adjacent to the atrial wall in epicardial fat pads, whereas sympathetic postganglionic somata are extracardiac. Transmural radiofrequency delivery from the endocardium can therefore attenuate parasympathetic input to the sinoatrial (SA) and AV nodes without epicardial access [6,7]. Subsequent single-center series, multicenter registries, and randomized trials have reported freedom-from-syncope rates of approximately 80–94% at 1–2 years in selected populations [8,9].

A major milestone was the 2024 joint scientific statement by the European Heart Rhythm Association (EHRA), Heart Rhythm Society (HRS), Asia Pacific Heart Rhythm Society (APHRS), and Latin American Heart Rhythm Society (LAHRS), which positioned CNA as a potential alternative to cardiac pacing in carefully selected patients [3]. Nevertheless, key questions remain unresolved: no large, blinded, sham-controlled trial has yet established the placebo-adjusted treatment effect; no adequately powered randomized head-to-head trial has determined whether CNA should be preferred to permanent pacing in patients older than 40 years with cardioinhibitory reflex syncope; no intraprocedural endpoint has been universally validated; the time course and clinical significance of reinnervation remain uncertain; and real-world results may be less favorable than outcomes from highly experienced centers [10,11].

Terminology remains an additional source of heterogeneity. Cardioneuroablation, cardioneural ablation, and ganglionated plexus ablation are often used interchangeably, although they may refer to different procedural strategies and endpoints. Recent calls for uniform nomenclature and outcome measures therefore reflect the need for a shared vocabulary [12]. In this review, CNA denotes endocardial catheter ablation of atrial ganglionated plexi aimed at parasympathetic cardiac denervation or neuromodulation. Accordingly, “ganglionated plexus ablation” is retained here only for the technical act of ablating a defined GP, most often in the context of atrial fibrillation, and “cardioneural ablation” only when citing sources that use that term.

This narrative review synthesizes current evidence on CNA for reflex syncope and functional bradyarrhythmias, with emphasis on neuroanatomy, diagnostic workup, patient selection, mapping and localization strategies, procedural endpoints, clinical outcomes, safety, and the ongoing randomized trials that will shape the next phase of the field.

## 2. Methods

The literature search was conducted in PubMed (https://pubmed.ncbi.nlm.nih.gov) and Web of Science (Clarivate, London, UK; https://www.webofscience.com), between 1 April and 31 May 2026. Rather than a single systematic query, searches were run section by section: for each thematic domain of the review, the terms “cardioneuroablation”, “cardioneural ablation”, and “ganglionated plexus ablation” were combined with domain-specific terms, including “vasovagal syncope”, “reflex syncope”, “atrioventricular block”, “sinus node dysfunction”, “ganglionated plexi”, “high-frequency stimulation”, “extracardiac vagal stimulation”, “computed tomography (CT)-guided fat pad segmentation”, “heart-rate variability”, “deceleration capacity”, and “vagal reinnervation”. Records were screened by title and abstract for relevance to the specific mechanistic, procedural, or clinical question under discussion, and the reference lists of key original studies, systematic reviews, and consensus documents were subsequently screened by hand to capture eligible articles not retrieved by the search terms. No date limit was applied, because the foundational anatomical and technical descriptions of the intrinsic cardiac nervous system remain directly relevant to contemporary practice.

Eligible sources addressed CNA or ganglionated plexus ablation for reflex syncope, functional sinus node dysfunction, or functional atrioventricular block in humans and were published in English; the study designs considered, and the weight assigned to each, are specified below. Two categories were excluded: isolated case reports, unless they illustrated a mechanistic or safety observation not documented elsewhere—for example, late functional AV block presenting after atrioventricular nodal reentrant tachycardia (AVNRT) ablation (Section 4.4)—and studies of ganglionated plexus ablation performed solely as an adjunct to pulmonary vein isolation for non-vagally mediated atrial fibrillation, unless directly informative for CNA technique or safety.

The 2024 EHRA/HRS/APHRS/LAHRS scientific statement and the 2023 EHRA clinical controversy document served as the principal reference frameworks [3,13]. Evidence was weighted according to a pre-specified hierarchy—randomized trials and international consensus or guideline documents first, followed by meta-analyses, multicentre prospective registries, and finally single-centre observational series—and, within each tier, preference was given to studies published in the past decade, to those reporting phenotype-specific outcomes, and to those providing procedural detail sufficient to judge reproducibility; the foundational sources noted above were exempt from this recency criterion. Where evidence was discordant, the conflict is presented rather than resolved. In the comparison between CNA and permanent pacing (Section 7.4), for example, randomized and blinded data were given precedence over non-randomized comparisons, and the specific limitations of the discordant studies—non-randomized design, phenotype imbalance between arms, and heterogeneous pacing modalities—are stated explicitly rather than averaged out. Consistent with a state-of-the-art narrative review, no Preferred Reporting Items for Systematic Reviews and Meta-Analyses (PRISMA) protocol, formal risk-of-bias assessment, or quantitative synthesis was undertaken; this thematic, non-systematic approach is the principal methodological limitation of the review and is addressed in Section 9.4. The objective was a mechanistically grounded synthesis for electrophysiologists, cardiologists, and syncope specialists that states explicitly where the current evidence is insufficient.

## 3. Physiological Rationale and Neuroanatomy

### 3.1. The Cardiac Autonomic Nervous System: From Armour to the Electrophysiology Laboratory

Cardiac rhythm and conduction are regulated not only by extracardiac autonomic inputs but also by an intrinsic neural network embedded in the heart itself. This system comprises sensory, local circuit, and motor neurons located predominantly in atrial and peri-great-vessel epicardial fat pads. Armour’s concept of the “little brain on the heart” remains the most useful physiological framework: intracardiac ganglia are not passive relay stations but an integrated neural network capable of processing afferent information and coordinating regional cardiac responses beat by beat [14].

The human anatomical substrate was defined by Armour and colleagues through systematic microdissection of human hearts. They described five major atrial ganglionated plexus (GP) regions, located predominantly on the posterior surfaces of both atria: the superior right atrial GP, the superior left atrial GP, the posterior right atrial GP, the posteromedial left atrial GP and the posterolateral left atrial GP. These five atrial regions provide the classical anatomical framework for CNA, although contemporary procedural strategies may further refine or expand them according to electroanatomical, imaging, and functional criteria. The human intrinsic cardiac nervous system was estimated to include more than 14,000 neurons distributed across approximately 550 ganglia, with the largest neuronal concentrations in the posterior right atrial and posteromedial left atrial regions [15].

A key implication is that each GP participates in a distributed network rather than acting as an isolated nodal relay: sensory, local circuit, adrenergic, and cholinergic neurons communicate continuously across all four chambers [14]. This sophistication explains both the physiological richness of cardiac autonomic regulation and the technical complexity of modifying it with focal endocardial ablation.

The translation of this anatomy to the electrophysiology laboratory depends on one practical fact: the atrial wall is thin, commonly on the order of a few millimeters, and GP are subepicardial structures embedded in adjacent fat pads. Radiofrequency energy delivered from the endocardial surface can therefore create transmural lesions capable of reaching these ganglia in selected regions, making catheter-based parasympathetic denervation feasible without surgical or epicardial access [3]. This anatomical proximity is the structural premise of CNA.

### 3.2. Ganglionated Plexus Nomenclature: Reconciling Anatomical, Electroanatomical, and Functional Systems

Several partially overlapping naming systems are used in the literature, and explicit reconciliation is essential. The classical anatomical nomenclature derived from Armour’s dissections uses location-based descriptors such as superior right atrial GP and posteromedial left atrial GP. Contemporary electroanatomical CNA practice more commonly uses procedural target labels: right superior GP (RSGP), right inferior GP (RIGP), left superior GP (LSGP), left inferior GP (LIGP), and posteromedial left GP (PMLGP) [3]. A simplified paraseptal nomenclature, increasingly used by European groups, emphasizes nodal innervation: the superior paraseptal GP (SPSGP), corresponding broadly to the RSGP, is a key final common pathway for right vagal input to the SA node; the inferior paraseptal GP (IPSGP), corresponding broadly to the PMLGP, is a key final common pathway for left vagal input to the AV node [13,16].

In addition to these five core atrial GP regions, extended contemporary CNA strategies may include two further autonomic targets: the Marshall tract GP (MTGP), located within the autonomic network of the Marshall complex along the left lateral ridge and left pulmonary venous carina; and the aorta–superior vena cava GP (Ao-SVC GP), a high right superior autonomic region located between the superior vena cava (SVC) and ascending aorta. These structures are not separate components of the original Armour five-region atrial classification; rather, they represent anatomically and procedurally relevant extensions that help bridge classical neuroanatomy, electroanatomical mapping, and imaging-guided CNA. Table 1 aligns these systems and clarifies the relationship between the five classical atrial GP regions and the expanded seven-target procedural framework.

### 3.3. The Cardioinhibitory Reflex Arc: From Trigger to Asystole

Cardioinhibitory reflex syncope reflects inappropriate activation of a neural circuit that normally maintains cardiovascular homeostasis. Afferent signals from mechanosensory and chemosensory neurites in the ventricles, atria, and great vessels are relayed to medullary cardiovascular centers, where orthostatic stress, emotional arousal, pain, or carotid sinus stimulation can trigger a paradoxical response—sympathetic withdrawal and abrupt parasympathetic activation—classically related to the Bezold–Jarisch reflex [3,14].

The efferent limb is transmitted through vagal preganglionic fibers arising from the nucleus ambiguus and dorsal motor nucleus of the medulla. These fibers synapse on postganglionic cholinergic neurons within atrial GP, particularly the RSGP/SPSGP for SA nodal modulation and the PMLGP/IPSGP for AV nodal modulation [3,15]. Additional GP regions, including the RIGP, LSGP, LIGP, MTGP, and Ao-SVC GP, contribute to the broader atrial autonomic network and may be incorporated into anatomical or phenotype-guided CNA strategies depending on the procedural approach. Acetylcholine release at muscarinic type 2 (M2) receptors in the SA and AV nodes then produces the clinical cardioinhibitory phenotype: sinus slowing or arrest, high-grade AV block, or both.

**Figure 1:**
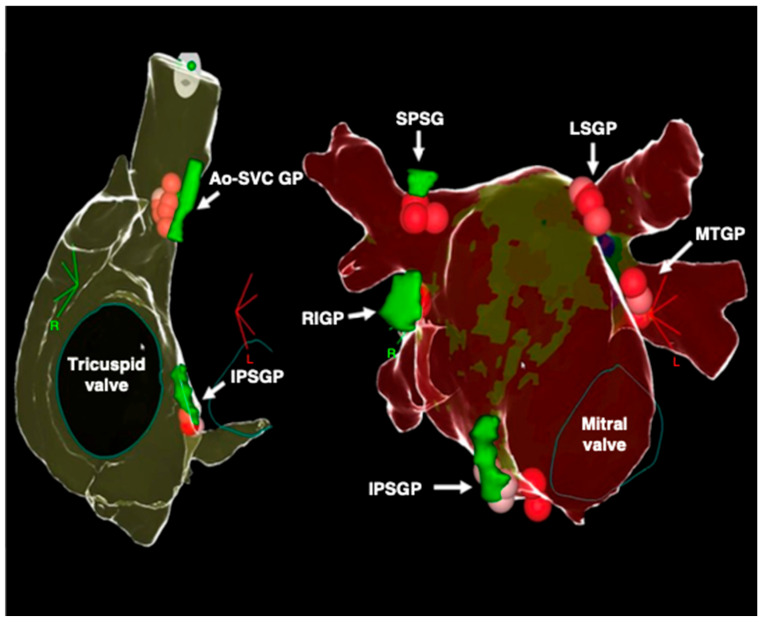
Anatomical targets in cardioneuroablation: the seven ganglionated plexus framework. Reproduced with permission from Gigante C et al. Europace. 2026;28(1):euaf320 [17]. Three-dimensional electroanatomical reconstruction showing the principal GP regions targeted during CNA, including the superior paraseptal GP (SPSGP), inferior paraseptal GP (IPSGP), right inferior GP (RIGP), left superior GP (LSGP), Marshall tract GP (MTGP), and aorto-superior vena cava GP (Ao-SVC GP). L, left; R, right.

CNA targets this final common efferent pathway. Because postganglionic parasympathetic neurons are located within or immediately adjacent to the atrial wall, whereas sympathetic postganglionic cell bodies are located in extracardiac ganglia, endocardial ablation can preferentially attenuate vagal input to the nodal tissues [3,16]. The therapeutic goal is not complete autonomic isolation of the heart but calibrated reduction in excessive vagal influence sufficient to prevent pathological cardioinhibition while preserving chronotropic reserve and broader autonomic responsiveness. Figure 1 summarizes the expanded seven-target anatomical framework commonly used to represent the principal GP regions relevant to contemporary CNA.

**Key points**

- The atrial GP constitute the parasympathetic component of the intrinsic cardiac nervous system, clustered in epicardial fat pads around the SA and AV nodal regions.
- Because the atrial wall is thin, endocardial radiofrequency can reach subepicardial GP, making catheter-based denervation feasible without epicardial access.
- The RSGP/SPSGP predominantly modulates SA nodal function, whereas the PMLGP/IPSGP predominantly modulates AV nodal function, providing the anatomical basis for phenotype-guided targeting.
- The five classical atrial GP regions described by Armour can be reconciled with an expanded seven-target contemporary CNA framework by incorporating the Marshall tract GP and the Ao-SVC GP as additional procedural targets.
- CNA interrupts the final common efferent limb of the vagal reflex at the postganglionic level; the aim is calibrated parasympathetic attenuation rather than complete autonomic isolation.

## 4. Who Is the Right Candidate? Indications and Patient Selection

Appropriate candidate selection is the dominant determinant of CNA success. Variability in published outcomes is explained at least as much by heterogeneity in patient phenotype as by differences in ablation technique. The optimal candidate has three core features: a documented cardioinhibitory mechanism, clinically significant symptoms despite adequate conventional management, and no evidence of structural heart disease or intrinsic conduction system disease. When these conditions coexist, CNA becomes a biologically coherent and clinically reasonable therapeutic option.

### 4.1. Identifying the Cardioinhibitory Phenotype

Phenotypic confirmation is the first essential step. Reflex syncope is not synonymous with cardioinhibitory syncope, and CNA has little mechanistic rationale in a purely vasodepressor phenotype, in which hypotension—not bradycardia or AV block—is the dominant driver of cerebral hypoperfusion [3,13].

Head-up tilt testing (HUTT) remains the most widely used phenotyping tool and allows classification according to the Vasovagal Syncope International Study (VASIS) classification. Type 2B responses (cardioinhibitory with asystole) and type 2A responses (cardioinhibitory without asystole, with heart rate below 40 bpm for more than 10 s) are the phenotypes with the strongest mechanistic rationale for CNA. By contrast, type 3 responses, in which the event is predominantly vasodepressor and heart rate does not fall substantially from its peak, should generally be considered unfavorable for CNA [3]. Even in apparent cardioinhibitory syncope, however, hypotension often precedes or accompanies bradycardia. The presence of asystole during HUTT should therefore not be interpreted as proof that the vasodepressor component is clinically irrelevant; residual vasodepression may persist, or become unmasked, after successful denervation [13].

Carotid sinus massage should be considered as a distinct provocative test, particularly in older patients, because it may identify a cardioinhibitory carotid sinus syndrome that overlaps with, but is not equivalent to, vasovagal syncope. Its diagnostic value is greatest when carotid sinus stimulation reproduces the patient’s symptoms in association with a significant cardioinhibitory pause. By contrast, implantable loop recorders (ILR) and other long-term monitoring strategies provide spontaneous symptom–rhythm correlation during real-life events, and therefore represent the strongest phenotypic evidence when they document symptomatic sinus arrest or high-grade AV block. HUTT, carotid sinus massage, and ILR should thus be interpreted as complementary but non-interchangeable tools: HUTT characterizes susceptibility to orthostatic/reflex provocation, carotid sinus massage identifies carotid sinus-mediated cardioinhibition, and ILR confirms the rhythm mechanism of spontaneous events. Because discordance between induced and spontaneous responses is common, candidate selection should integrate clinical history, provocative testing, and objective rhythm documentation whenever possible [3].

**Figure 2:**
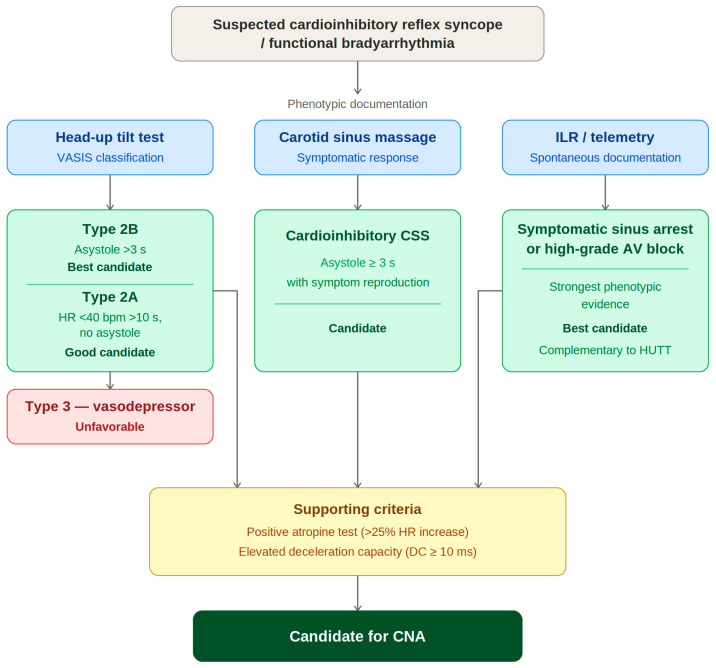
Diagnostic pathway for identification of the cardioinhibitory phenotype and candidate selection for cardioneuroablation. CNA, cardioneuroablation; CSS, carotid sinus syndrome; DC, deceleration capacity; HR, heart rate; ILR, implantable loop recorder; VASIS, Vasovagal Syncope International Study.

Cardiac deceleration capacity (DC) has emerged as a promising quantitative marker of heightened vagal tone. In patients with refractory vasovagal syncope (VVS), elevated nighttime DC identified individuals with greater benefit from CNA, and each 1 ms increase in DC was associated with a lower recurrence risk [18]. In a separate cohort, DC was used to select patients with paroxysmal atrial fibrillation (AF) for autonomic ablation, with favorable outcomes when procedural heart-rate acceleration confirmed denervation [19]. These findings support DC as a continuous biomarker that may complement the binary cardioinhibitory/vasodepressor classification, although prospective validation as an enrollment criterion remains necessary. Figure 2 summarizes the resulting diagnostic pathway for confirming the cardioinhibitory phenotype and selecting candidates for CNA.

### 4.2. Clinical Impact: When Syncope Becomes Dangerous

Episode frequency alone should not determine candidacy for an invasive procedure. The clinical relevance of syncope depends on unpredictability, absence of prodrome, traumatic consequences, occupational risk, driving implications, and the psychological burden of anticipation. A single episode causing injury or occurring while driving may justify a more aggressive strategy than multiple benign episodes with reliable prodrome and safe recovery [20].

Van Dijk and colleagues emphasized that invasive therapy should be considered only after an adequate trial of conventional management—education, lifestyle modification, trigger avoidance, hydration, counterpressure maneuvers, and pharmacotherapy where appropriate—given the high spontaneous remission and placebo-response rates of VVS [20,21].

The most compelling scenarios for CNA are recurrent syncope without sufficient warning, syncope occurring in high-risk circumstances, or episodes causing physical injury, provided a dominant cardioinhibitory mechanism has been confirmed and intrinsic conduction disease has been excluded.

### 4.3. Age Matters: From the Elderly to the Pediatric Patient

#### 4.3.1. The Older Patient

Guidelines on syncope and cardiac pacing support dual-chamber pacing in selected patients, generally older than 40 years, with documented cardioinhibitory reflex syncope. Pacing therefore remains the conventional evidence-based option in this group, whereas CNA has not yet received a formal guideline recommendation. Nevertheless, CNA is mechanistically attractive for patients—particularly those aged 40–60 years—with a clear cardioinhibitory phenotype, a structurally normal heart, and a strong preference to avoid lifelong device therapy [3,13].

The ELEGANCE multicenter study examined the effect of age on CNA outcomes and reported overall freedom from symptoms of 88%, without statistically significant differences between age strata, although efficacy tended to be lower in patients older than 60 years [22]. In a large prospective single-center series of 209 patients followed for a median of 27 months, age was not an independent predictor of efficacy; however, recurrence was more frequent in older patients, and all patients who ultimately required pacemaker implantation after CNA failure were older than 55 years [23]. A prospective single-center study comparing patients younger and older than 50 years found comparable syncope recurrence, pacemaker-free survival, and degree of denervation assessed by heart-rate variability and atropine testing across both age groups, supporting the view that selection should be guided by autonomic functional testing rather than by a fixed age threshold [24]. Thus, age above 60 years is not an absolute contraindication, but it should prompt careful assessment for intrinsic conduction disease and more guarded counseling.

#### 4.3.2. The Pediatric Patient

At the other end of the age spectrum, CNA addresses a difficult pediatric problem: functional asystolic pauses in young patients for whom the standard alternative may be pacemaker implantation with repeated generator changes, lifelong lead-related risk, and activity restrictions [25]. CNA offers the possibility of treating the autonomic mechanism and avoiding permanent hardware.

Choi and Moak described six patients aged 21 years or younger with functional sinus pauses or paroxysmal AV block in whom the median longest pause decreased from 8.9 to 1.1 s at 6 months, with no recurrent syncope after the procedure [26]. A Spanish single-center pediatric series from Sant Joan de Déu Hospital included 10 patients (mean age 13.8 years, range 11–17) treated with near-zero fluoroscopy biatrial CNA guided by anatomical and fractionated electrogram criteria. The longest pre-CNA pause was 20 s; at 6 months, the mean longest pause was 1.1 s, with no documented pauses and no complications [27]. These early data support feasibility but should be interpreted cautiously until larger pediatric registries with standardized follow-up are available.

### 4.4. Functional Bradyarrhythmias Beyond Vasovagal Syncope

Although CNA was initially developed for vasovagal syncope, the same anatomical logic applies to functional bradyarrhythmias caused by excessive parasympathetic input to the SA or AV node in the absence of intrinsic conduction disease.

Functional, vagally mediated AV block is characterized by paroxysmal high-grade supra-Hisian block in a structurally normal heart, preserved infra-Hisian conduction, and reversibility with atropine. The PIRECNA multicenter registry supports CNA as a potential pacemaker-sparing option in this setting [28]. The main anatomical target is the PMLGP/IPSGP, which provides dominant vagal input to the AV node, and a useful procedural endpoint is normalization or clear shortening of the antegrade Wenckebach point after ablation in this region [29].

A practical illustration comes from Bellvitge University Hospital, where CNA was used in three patients who developed paroxysmal complete AV block 97–127 months after successful AVNRT ablation, a recognized but uncommon late complication. In all cases, normal infra-Hisian conduction and preserved atropine response supported a functional mechanism. CNA targeting the PMLGP and LSGP achieved acute procedural success, with no recurrent syncope at 6–13 months [30]. This experience suggests that AVNRT ablation may, in selected patients, unmask a pre-existing vagal susceptibility by modifying slow-pathway physiology, and that CNA can address the autonomic component without immediate pacemaker implantation.

Functional sinus node dysfunction follows the same principle. A robust heart-rate increase after atropine, usually exceeding 25–30% without evidence of AV block, indicates preserved intrinsic nodal reserve and a predominantly vagal mechanism [23,29]. In contrast, a blunted atropine response, structural heart disease, or progressive conduction abnormalities should redirect management away from CNA and toward conventional bradycardia evaluation and pacing when appropriate.

### 4.5. The Athlete’s Heart: A High-Vagal-Tone Phenotype

Endurance athletes constitute a distinctive high-vagal-tone population. Sinus bradycardia, first-degree AV block, Mobitz I second-degree AV block, and nocturnal sinus pauses are common in highly trained individuals and are often considered physiological adaptations [31].

However, athletic bradycardia is not always purely autonomic. Experimental and clinical evidence indicates that intrinsic sinus-node remodeling, including changes in hyperpolarization-activated cyclic nucleotide-gated channel 4 (HCN4) expression and pacemaker current, may contribute independently of vagal tone [31]. This distinction is clinically decisive: CNA is unlikely to benefit an athlete whose bradycardia is primarily due to intrinsic nodal remodeling. The atropine test therefore becomes central: a brisk chronotropic response supports a functional vagal mechanism, whereas a blunted response should raise concern for intrinsic nodal disease or maladaptive remodeling.

Clinical evidence in athletes remains limited to case reports and small series. The 2024 HRS expert consensus statement recognizes CNA as a technically plausible option for selected symptomatic bradyarrhythmias but stops short of a formal recommendation owing to the absence of controlled outcome data [25]. In older athletes, intrinsic nodal disease may coexist with high vagal tone, making the atropine test particularly decisive.

**Key points:**

- CNA should be reserved for patients with a documented predominantly cardioinhibitory mechanism and clinically important symptoms despite appropriate conventional management [3,13].
- Spontaneous symptom–rhythm correlation obtained with an implantable loop recorder provides the strongest phenotypic evidence; head-up tilt testing and carotid sinus massage are complementary rather than interchangeable.
- A dominant vasodepressor response, a blunted atropine response, structural heart disease, or progressive conduction disease argues against CNA.
- In functional sinus node dysfunction or atrioventricular block, preserved atropine responsiveness and normal infra-Hisian conduction support a vagally mediated substrate.
- Age alone should not determine eligibility, but older patients require particularly rigorous exclusion of intrinsic conduction disease and balanced discussion of guideline-supported pacing.

## 5. Tailored Ganglionated Plexus Targeting for Cardioneuroablation

The central technical question in CNA is not only where to ablate, but also how much autonomic tissue should be modified and how success should be confirmed. The field has moved from broad empirical ablation of all accessible GP toward a more selective, phenotype-guided strategy. This evolution is clinically important: smaller and better-targeted lesion sets may reduce procedure time, collateral injury, and excessive denervation while preserving efficacy.

### 5.1. Phenotype-Guided Target Selection

The anatomical basis for tailored targeting is the partial lateralization of vagal control: the right vagus exerts dominant influence on the SA node through the RSGP/SPSGP, whereas the left vagus exerts dominant influence on the AV node through the PMLGP/IPSGP [3,13]. This asymmetry is not absolute because interganglionic connections are extensive, but it is consistent enough to guide strategy. Ablation of the SPSGP typically increases sinus rate, indicating SA nodal denervation; ablation of the IPSGP shortens AV nodal refractoriness and may increase the antegrade Wenckebach point, indicating AV nodal denervation. Responses from the LSGP, LIGP, and RIGP are more variable and usually less specific. The aorto-caval GP and Marshall tract GP are also less consistently targeted, but may contribute to the autonomic substrate in selected patients [3].

A practical issue is sequence dependence. Because vagal inputs converge through interconnected GP, early ablation of right-sided targets can attenuate or abolish responses that would otherwise identify left-sided plexi. For this reason, many operators either confirm all candidate targets with high-frequency stimulation before ablation or begin with left-atrial/inferoseptal targets before proceeding to right-sided sites [32]. The order of ablation should therefore be considered part of the diagnostic strategy, not merely a technical preference.

The response observed during radiofrequency delivery provides additional functional confirmation. At the RSGP/SPSGP, ablation usually produces a prompt and sustained increase in sinus rate, sometimes after an initial transient vagal reaction. At the LSGP, a brief vagal response followed by acceleration is common. At the PMLGP/IPSGP and inferior paraseptal region, the most relevant sign is progressive PR-interval shortening, improved AV conduction, or resolution of vagally mediated AV block during energy delivery [31,33].

The first prospective multicenter comparison of a phenotype-guided tailored strategy with comprehensive all-GP ablation was reported by Gigante, Berruezo, San Antonio, and colleagues in the ELEGANCE study. Among 123 patients treated at five European centers, 54 underwent a tailored protocol targeting the SPSGP for isolated functional sinus node dysfunction, the IPSGP for isolated functional AV block, and both targets for mixed phenotypes; 69 underwent standard biatrial ablation of all GP. The tailored group required only 1.6 ± 0.7 GP targets per patient. Acute procedural success was similar (93% vs. 90%; *p* = 0.98), syncope-free survival at a median 15.9 months was comparable (log-rank *p* = 0.96), and the tailored strategy significantly reduced procedure time (63 vs. 85 min; *p* = 0.001) and radiofrequency (RF) time (5.4 vs. 10.4 min; *p* < 0.001) [17].

These findings shift the debate from feasibility to proportionality. If ablation confined to phenotype-relevant GP provides equivalent medium-term outcomes, extensive biatrial denervation may not be necessary in many patients. A tailored approach may preserve autonomic reserve while suppressing the pathological reflex. Whether this advantage persists after longer follow-up and possible reinnervation remains a key testable hypothesis.

### 5.2. The Left-Atrial-First and Biatrial Debate

ROMAN2 randomized 40 patients with reflex asystolic syncope to right-atrial-first versus left-atrial-first CNA, using extracardiac vagal stimulation (ECVS) as the intraprocedural endpoint. Complete vagal denervation was achieved in 35% of patients after right-atrial-only ablation compared with 80% after left-atrial-only ablation (*p* = 0.01). The right-atrial approach was particularly ineffective for AV nodal denervation, requiring crossover to the left atrium in 65% of cases [16].

The randomized trial by Tu et al. compared left-atrial-only with biatrial CNA in 80 patients with VVS in a non-inferiority design. At 12 months, the left-atrial approach was non-inferior to biatrial ablation for freedom from recurrent syncope (87.5% vs. 90%; *p* = 0.723; *p* for non-inferiority = 0.001), while reducing procedure time, X-ray dose, lesion number, and RF time [34].

Taken together, these data support a left-atrial-first, phenotype-guided strategy, with right-atrial extension reserved for cases in which the intended functional endpoint is not achieved. This approach may reduce procedural burden and excessive right-sided denervation without compromising efficacy.

### 5.3. Ganglionated Plexus Localization Techniques

No GP localization method has been universally accepted, and most contemporary procedures integrate several complementary layers. The main strategies are summarized in Table 2.

### 5.4. Anatomical Targeting

The most reproducible strategy is anatomical targeting of regions known to harbor major GP: the anterior antrum of the right superior pulmonary vein for SPSGP/RSGP, the inferior–posterior left atrium near the coronary sinus ostium for IPSGP/PMLGP, and the left atrial ridge for LSGP. Anatomical targeting requires no dedicated equipment beyond a standard electroanatomic mapping system and aligns with consensus recommendations [3]. Its limitation is biological variability: GP position, density, and endocardial accessibility differ between patients, so anatomy alone may both miss active plexi and expose non-relevant tissue to unnecessary ablation.

### 5.5. High-Frequency Stimulation

High-frequency stimulation (HFS) tests the functional activity of candidate GP sites by delivering rapid pacing trains and observing for vagal responses such as sinus slowing, asystole, or AV block. Protocols vary, but commonly include continuous trains at 10–20 Hz for several seconds or synchronized stimulation protocols used to detect ectopy-triggering autonomic sites. A positive response is generally defined by marked R-R prolongation, sinus bradycardia, or AV block during stimulation [35,36]. HFS provides functional confirmation and is particularly useful when anatomical and electrogram-based markers are ambiguous.

HFS has important limitations: stimulation can induce AF, responses are sedation-dependent, and atropine renders subsequent HFS unreliable. The absence of a vagal response does not exclude nearby autonomic tissue if stimulation intensity is insufficient or the target is deeply embedded. HFS is therefore best used as a confirmatory layer rather than the sole mapping strategy [37,38].

### 5.6. Fractionated Electrogram Analysis

Fractionated electrogram (fEGM) mapping is based on the observation that neural fibers and heterogeneous tissue architecture overlying GP may produce multicomponent local electrograms during sinus rhythm. High-density mapping with dedicated software can display areas of electrogram fragmentation and guide ablation. For reproducibility, acquisition settings are critical: contemporary workflows typically use high-density multielectrode catheters with band-pass filtering around 200–500 Hz and define fractionation by deflection count and software-specific parameters [36,39,40]. The major caveat is lack of specificity: scar, fibrosis, anisotropic conduction, and far-field signals can also produce fractionated electrograms. fEGM maps should therefore be interpreted in anatomical and functional context.

### 5.7. Spectral and Peak-Frequency Analysis (OTNF)

The original CNA technique used spectral analysis to distinguish compact myocardium from fibrillar, GP-associated myocardium based on dominant frequency characteristics. A contemporary implementation is the Omnipolar Technology Near Field (OTNF) algorithm in EnSite X (Abbott, Abbott Park, IL, USA), which annotates the highest peak frequency of bipolar or omnipolar electrograms and helps separate sharp near-field activity from rounded far-field components [41].

For CNA, high peak-frequency regions around anatomical GP locations, often using thresholds near 600 Hz after construction of a conventional fractionation map, have been used to refine target localization [46]. Importantly, the highest-frequency zones do not always coincide with the most fractionated zones. This spatial discordance suggests that peak-frequency and fractionation mapping provide complementary, not interchangeable, information [41,42].

### 5.8. CT-Guided Fat-Pad Segmentation

Pre-procedural cardiac CT can identify epicardial fat pads using standard Hounsfield unit thresholds (typically −190 to −30 HU). Segmentation of these fat pads and integration into the electroanatomic map, for example with ADAS 3D (ADAS 3D Medical SL, Barcelona, Spain) or inHEART (inHEART, Pessac, France), provides an anatomical scaffold for GP targeting. Francia et al. showed that the SPSGP had the highest rate of RF response when ablated directly over the corresponding fat pad (84%), supporting its anatomical consistency as a target for SA nodal denervation. Across all GP, most RF-responsive sites were located directly over the segmented fat pad, or only a few millimeters from it [44].

However, fat is a surrogate for autonomic tissue, not a direct marker of neuronal activity. Benabou et al. reported weak spatial correlation between epicardial fat pads and fEGM-defined GP in the left atrium and even negative correlation in the right atrium [4]. Thus, CT integration improves anatomical awareness and procedural planning but should not be used in isolation, and is best applied in combination with other techniques such as fEGM or OTNF-based mapping.

### 5.9. Intracardiac Echocardiography (ICE)-Guided Ablation

Intracardiac echocardiography (ICE) can visualize paraseptal fat pads in Waterston’s groove and the pyramidal space in real time, allowing right-sided CNA guided by direct anatomical landmarks. Farwati et al. reported feasibility in 17 patients with recurrent cardioinhibitory VVS referred for pacemaker implantation: target fat pads were identified in all patients and reconstructed in the electroanatomic map within minutes, and 94% remained free from recurrent symptoms at 12 months [45]. An important safety advantage of ICE is visualization of the right phrenic nerve as a hyperechoic linear structure near the posterolateral superior vena cava, enabling its reconstruction and exclusion from the ablation field.

### 5.10. Combining Multiple Strategies

The complementary value of combining multiple localization strategies was examined in a single-center retrospective study from Bellvitge University Hospital [43]. In 10 patients undergoing CNA, 237 radiofrequency applications (46 with positive response, 191 without) were analyzed according to four concurrently applied strategies: empirical anatomical reference, CT-segmented epicardial fat pads using the Anatomical Detection and Ablation System 3D (ADAS 3D), fractionated electrogram mapping, and automated peak-frequency analysis (OTNF ≥ 450 Hz, EnSite X (Abbott, Abbott Park, IL, USA)).

Among applications with a positive response—defined as heart-rate acceleration, vagal response, or PR shortening >10% sustained for >10 s—87% showed convergence of at least three strategies in the same area, compared with 42% among non-responding applications. Convergence of all four strategies was present in 41% of positive-response sites versus 12% of negative-response sites. The number of coincident methods was a significant predictor of response (area under the receiver-operating-characteristic curve [AUC] 0.765); a threshold of three or more converging strategies provided sensitivity of 87% and specificity of 58%.

**Figure 3:**
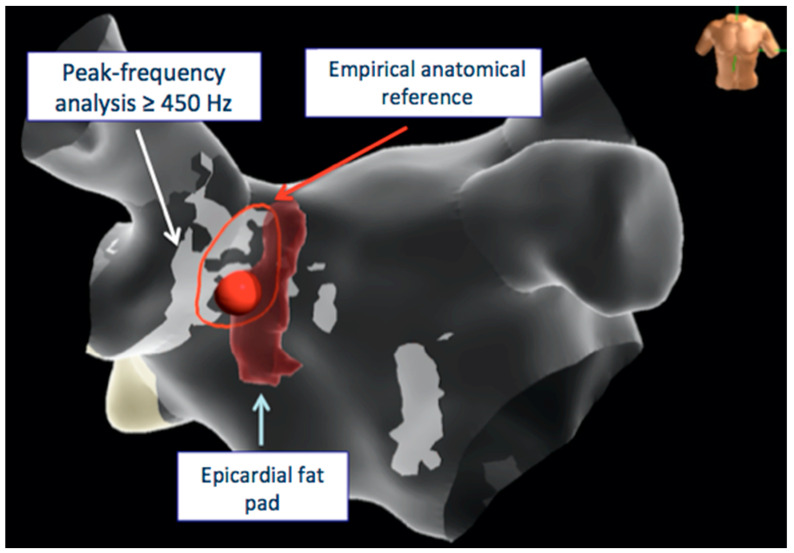
Multimodal integration of GP localization strategies: convergence of empirical anatomical reference, CT-segmented epicardial fat pads (ADAS 3D), and peak-frequency analysis (OTNF ≥ 450 Hz) on an electroanatomic map (EnSite X).

These findings support a multimodal integration approach: targeting areas where anatomical, electrographic, spectral, and imaging evidence align may improve procedural efficiency and reduce unnecessary radiofrequency applications, although prospective validation in larger cohorts is required. Figure 3 illustrates this convergence of localization strategies on an electroanatomic map.

### 5.11. Radiofrequency Parameters

Reported radiofrequency settings vary according to target location and local wall thickness. Lesions are usually delivered point by point, using contact force around 10–20 g, power around 40 W, and ablation index values of 450–550 (lesion index 5.0–6.0), with more conservative settings near the posterior coronary sinus ostium and other vulnerable structures, particularly the sinus node artery, AV nodal inputs, phrenic nerve, esophagus, and venous structures [35,46]. Practical guidance on titrating contact force and power according to anatomical site and catheter stability has been summarized in a dedicated review [46]. The relevance of local wall thickness to lesion transmurality has been demonstrated in a prospective multidetector CT-guided study of persistent atrial fibrillation ablation, in which ablation index was individualized according to left atrial wall thickness at each point; regions without first-pass isolation and reconnection sites at redo procedures were predominantly the thickest antral segments [47]. Although that experience concerns pulmonary vein isolation rather than CNA, the principle is directly relevant to atrial GP targets, where wall thickness varies substantially between sites. Radiofrequency duration should also be guided by the physiological response observed during ablation, with repeat applications at the same site if a vagal or conduction response persists.

**Key points:**

- Phenotype-guided targeting can reduce lesion number, procedure duration, and radiofrequency time without compromising medium-term outcomes in appropriately selected patients [17].
- A left-atrial-first strategy is generally more efficient; right-atrial extension should be driven by failure to achieve the intended functional endpoint [16,34].
- No single localization technique provides both complete anatomical definition and functional validation; concordance between complementary methods increases confidence but is not itself a validated procedural endpoint.
- Mapping sequence matters: candidate sites should ideally be characterized before ablation and before atropine, because prior denervation can attenuate subsequent vagal responses.
- Lesion delivery should account for local wall thickness and proximity to the sinus node artery, atrioventricular nodal inputs, phrenic nerve, esophagus, and venous structures.

## 6. How Do We Know It Worked? Procedural Endpoints

Unlike pulmonary vein isolation or accessory pathway ablation, CNA does not produce a binary electrical endpoint such as entrance block, exit block, or elimination of a discrete pathway potential. Success must be inferred from physiological evidence of parasympathetic attenuation. Defining a reproducible, outcome-linked procedural endpoint is therefore one of the most important unresolved issues in the field [48].

### 6.1. Acute Endpoints in Current Use

Current endpoints attempt to document effective parasympathetic attenuation at either a global or target-specific level. Their strengths and limitations are summarized in Table 3.

Sedation depth is an important confounder for all vagal response-based endpoints. Responses elicited during GP stimulation or ablation may differ substantially between conscious sedation and deep sedation or general anesthesia. A sustained postprocedural increase in resting heart rate has been associated with freedom from syncope and with negative repeat HUTT, supporting its value as a simple surrogate of denervation. However, because heart rate is non-specific and sensitive to baseline autonomic state, it should be interpreted alongside target-specific endpoints [50].

### 6.2. Post-Procedural Verification and Follow-Up

Follow-up after CNA should combine symptom assessment with objective rhythm and autonomic monitoring. Resting heart rate, ambulatory electrocardiographic (ECG) monitoring, heart-rate variability (HRV) indices, and, in selected high-risk patients, ILR data provide complementary measures of denervation durability and recurrent asystolic burden. Repeat HUTT may be informative in selected cases, but it should be interpreted cautiously because tilt responses may not mirror spontaneous events [51].

**Figure 4:**
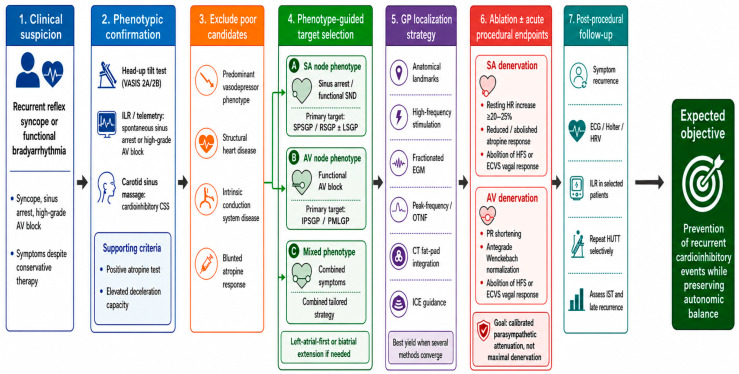
Practical algorithm for patient selection and procedural strategy in cardioneuroablation. The workflow proceeds from clinical suspicion (recurrent reflex syncope or functional bradyarrhythmia despite conservative therapy) through phenotypic confirmation (head-up tilt testing, implantable loop recorder or telemetry, carotid sinus massage, supported by a positive atropine test and elevated deceleration capacity) and explicit exclusion of poor candidates (predominant vasodepressor response, structural heart disease, intrinsic conduction system disease, blunted atropine response), to phenotype-guided target selection (sinoatrial, atrioventricular, or combined phenotype), ganglionated plexus localization strategy, ablation with confirmation of acute procedural endpoints, and post-procedural follow-up. Localization yields most when several methods converge, and the procedural goal is calibrated parasympathetic denervation rather than maximal denervation.

A central balance must be maintained between complete and partial denervation. More extensive ablation may achieve a stronger acute endpoint but may also increase the risk of inappropriate sinus tachycardia, excessive HRV suppression, and uncertain long-term autonomic effects. Conversely, overly conservative ablation may be more susceptible to reinnervation or incomplete clinical effect. The ELEGANCE tailored data suggest that, in phenotypically well-defined patients, functional denervation of the relevant nodal target may matter more than anatomical coverage of every GP [17]. Figure 4 integrates this phenotype-guided logic into a practical clinical workflow, from candidate selection to post-procedural surveillance.

**Key points:**

- Procedural endpoints should match the treated phenotype: sinus-rate and atropine responses for sinoatrial denervation, PR/Wenckebach changes for atrioventricular nodal denervation, and extracardiac vagal stimulation for global functional assessment.
- A post-ablation increase in resting heart rate is simple and reproducible but non-specific and should not be used as a stand-alone endpoint.
- Extracardiac vagal stimulation is the most direct functional test of denervation, although its use remains limited by equipment, expertise, and protocol standardization.
- Atropine testing is best reserved for the end of the ablation sequence because its administration prevents reliable repetition of vagal-response mapping.
- Follow-up should integrate symptoms with electrocardiographic, heart-rate variability, and, when available, implantable-loop-recorder data; late recurrence after an initial response should raise the possibility of vagal reinnervation.

## 7. Clinical Evidence: What Do We Know So Far?

The CNA evidence base has advanced rapidly. In less than two decades, the field has moved from proof-of-concept series to randomized clinical trials, multicenter registries, and multiple systematic reviews and meta-analyses. This trajectory is unusual for a procedure aimed at a condition traditionally treated conservatively or with pacing, and it reflects both the unmet need and the strength of the mechanistic rationale.

### 7.1. Randomized Controlled Trials

**ROMAN: the first randomized evidence**

The ROMAN trial remains the pivotal randomized evidence for CNA efficacy [8]. It enrolled 48 patients (mean age 38 ± 10 years, 58% female) with highly symptomatic cardioinhibitory or mixed VVS, failure of non-pharmacological treatment, and a positive atropine test. Patients were randomized 1:1 to biatrial CNA or continued optimized non-pharmacological management.

At 2 years, recurrent syncope occurred in 2 of 24 patients in the CNA group (8%) compared with 13 of 24 controls (54%; *p* = 0.0004). CNA also produced coherent autonomic and electrophysiological changes: immediate sinus-rate acceleration, shortening of corrected sinus-node recovery time, improved AV nodal conduction, and marked attenuation of the atropine response. Quality of life improved substantially in the CNA group, and most control patients who crossed over to CNA after study completion remained syncope-free during subsequent follow-up [8].

The limitations of ROMAN are important. The trial was open-label, the control arm did not include sham intervention or pharmacological therapy, and ECVS was not used as a procedural endpoint. Nevertheless, ROMAN established a randomized efficacy signal that is directionally consistent with the expected autonomic effects of CNA and remains the reference trial for the field.

**Tu et al.: left vs. biatrial CNA**

The randomized trial by Tu et al. addressed the optimal extent of ablation by randomizing 80 patients with VVS to left-atrial-only or biatrial CNA [34]. At 12 months, left-atrial ablation was non-inferior to biatrial ablation for freedom from recurrent syncope (87.5% vs. 90%), while reducing procedure time, X-ray dose, lesion number, and RF time. This trial supports the concept that a less extensive strategy can preserve efficacy when the dominant autonomic substrate is adequately targeted.

### 7.2. Meta-Analyses and Systematic Reviews

Systematic reviews and meta-analyses now synthesize data from more than 2000 patients. Across these analyses, the recurrent syncope rate after CNA is consistently low, corresponding to approximately 80–94% freedom from syncope at medium-term follow-up. This convergence is encouraging but must be interpreted in light of heterogeneous populations, variable procedural endpoints, and limited sham-controlled evidence.

Vandenberk et al. performed the first comprehensive meta-analysis of 465 patients across 14 studies [2]. Pooled freedom from syncope was 91.9% (95% confidence interval (CI) 88.1–94.6%). Right-atrial-only ablation was significantly inferior to LA-only or biatrial approaches (81.5% vs. 94.0% vs. 92.7%; *p* < 0.001), whereas the GP localization technique did not influence efficacy.

Armani Prata et al. updated the evidence base with 1,153 patients from 27 observational studies plus the ROMAN randomized controlled trial (RCT) [9]. The weighted syncope recurrence rate was 5.94% (95% CI 3.37–9.01%), with a periprocedural complication rate of 0.99% (95% CI 0.14–2.33%). More recent meta-analyses have extended the field to network comparisons against pacing and pharmacological therapy and to functional bradyarrhythmias beyond VVS, supporting a consistent efficacy signal while reinforcing the need for higher-quality randomized data [52,53].

### 7.3. Key Registries and Multicenter Studies

The most influential randomized trials, multicentre registries, and meta-analyses underpinning current practice are summarized in Table 4, which sets out study design, sample size, patient phenotype, ablation strategy, follow-up duration, and the reported recurrence and major adverse event rates for each.

#### 7.3.1. Large International Registries

Large registries have expanded the field beyond single expert centers. PIRECNA focused on functional AV block and supported CNA as a pacemaker-sparing option in structurally normal hearts with documented supra-Hisian block and atropine responsiveness [28]. Across 130 patients from 20 centres, acute procedural success was 96.2% and 77.3% remained free of syncope or recurrent daytime second- or advanced-degree AV block at 360 days, with only four patients requiring a pacemaker and no major procedure-related complications (Table 4) [28]. The ELEGANCE program has contributed multicenter European data on imaging integration, age, atrial-side contribution, and phenotype-guided tailored ablation [17,22,32,44].

The United States Multicenter CNA Registry is the largest real-world experience published to date, including 205 patients from 15 centers between 2018 and 2024 [11]. At a mean follow-up of 14 ± 11 months, 78% of patients with syncope remained free from recurrence, and 97% avoided pacemaker implantation. However, complications occurred in 4.7% of procedures, including sinus node dysfunction, hemopericardium, right diaphragmatic paralysis, and death, with a 1.4% major adverse event rate. These data underscore that outcomes outside expert centers may differ materially from the most favorable single-center reports. The CNA-FWRD registry is a prospective multicentre registry with a cross-over design that will compare conservative therapy with CNA across VVS, sinus bradycardia, and functional AV block over three years of follow-up; its protocol has been published, but no outcome data are yet available [35].

#### 7.3.2. Predictors of Success: The Barrio-López Multicenter Study

Barrio-Lopez et al. reported a Spanish multicenter experience of 77 consecutive patients treated at 22 hospitals, a cohort reflecting early real-world dissemination [10]. At a median follow-up of 12 months, 66.2% remained free from syncope, lower than the 88–94% reported in many expert-center series. Predictors of success included male sex, older age, cardioinhibitory rather than mixed phenotype, deep sedation or general anesthesia, and a higher number of RF applications. The finding that all rhythm complications occurred in women warrants attention and further investigation.

Upadhyay’s accompanying editorial framed these results as a call for technical and mechanistic refinement rather than indiscriminate expansion of indications [54]. The broader lesson is that CNA is highly operator- and phenotype-dependent: reproducible targets, procedural endpoints, and training standards must be defined before widespread adoption can be considered mature.

### 7.4. CNA Versus Pacemaker: A Head-to-Head Perspective

For clinical electrophysiologists, the practical question is whether CNA can replace or delay pacemaker implantation in selected patients with cardioinhibitory VVS and documented asystole. The only direct comparative data currently come from a non-randomized multicenter study by Gopinathannair et al. including 162 patients with cardioinhibitory VVS (61 CNA and 101 pacemaker) across five centers [55]. At 1 year, freedom from syncope was numerically higher after CNA than after pacemaker implantation (97% vs. 89%), but the adjusted difference did not reach statistical significance.

An accompanying commentary on this study argued that its design may have systematically disadvantaged the pacing arm: the pacing group included a significantly higher proportion of patients with a mixed cardioinhibitory–vasodepressor response, even though guidelines restrict pacing to the cardioinhibitory phenotype, and pacing modalities were heterogeneous (closed-loop stimulation (CLS), rate-drop response, and leadless pacing). This heterogeneity may understate the benefit reported with dual-chamber closed-loop pacing in dedicated trials such as SPAIN and BioSync CLS [56,57]. The same commentary noted that the relatively young study population (mean age 35.6 ± 10 years) limits direct comparison with the older cohorts in which most pacing evidence has been generated, that quality of life was not formally assessed, and that an adequately powered randomized trial directly comparing CNA with pacing is still needed [58]. Taken together, these caveats mean that the numerical advantage observed with CNA cannot be read as equivalence, let alone superiority. Permanent pacing remains the only guideline-endorsed therapy for cardioinhibitory reflex syncope in patients meeting standard criteria, supported by decades of clinical experience and by the dedicated randomized trials noted above (Table 5).

The mechanistic argument for CNA is strongest in younger patients. Pacing treats the bradycardic endpoint of the reflex but does not modify the autonomic trigger and does not address vasodepression. It also creates lifelong device dependency in patients who may otherwise have structurally normal hearts. CNA, in contrast, attenuates efferent vagal input to the SA and AV nodes and may therefore prevent cardioinhibition at its source. Its role, on current evidence, is therefore not to replace pacing across the eligible population but to offer a complementary, mechanism-directed and device-sparing option to carefully selected younger patients who wish to avoid an implant—a position that will stand or fall on PANACEA, the adequately powered randomized comparison of the two strategies now under way (Section 9.1) [59].

### 7.5. The Sham-Control Debate

The most important methodological criticism of the CNA literature is the absence of completed sham-controlled efficacy trials. Chakraborty et al. argued that, given the high spontaneous remission and placebo response of VVS, open-label outcomes cannot separate the specific effect of ablation from regression to the mean, procedural attention, and expectation, and they highlighted the potential risks of excessive parasympathetic withdrawal, including proarrhythmic vulnerability [39]. Wichterle and Kulakowski replied that this critique underestimates the coherence of the sustained changes in heart rate, HRV, and atropine response that follow CNA—changes a placebo response would not be expected to produce—while conceding that persistent resting tachycardia after denervation may carry long-term cardiovascular implications, which argues for calibrated neuromodulation rather than maximal denervation [61].

The practical consequence is that neither the 80–94% freedom from syncope reported across open-label series nor the treatment effect observed in ROMAN can currently be partitioned into a specific, ablation-related component and a non-specific one. Reflex syncope is unusually susceptible to this ambiguity: patients are enrolled at a symptomatic peak, the natural history is one of spontaneous remission, and the intervention is an invasive procedure accompanied by sustained clinical attention [20,21,61]. At present, therefore, the specific efficacy of CNA is better described as plausible and physiologically supported than as established.

This is not a hypothetical concern, because the field has already run this experiment once. Permanent pacing for vasovagal syncope followed an almost identical trajectory: three open-label randomized trials reported that pacing markedly reduced syncope, and the therapy entered practice on that basis. When the question was reasked under double-blind conditions, comparing pacemaker ON with pacemaker OFF in patients who had all been implanted, the effect largely disappeared—VPS II found no significant reduction in recurrent syncope, and SYNPACE could not demonstrate superiority of active over inactive pacing [62,63]. A meta-analysis of nine randomized trials subsequently quantified the discrepancy: awareness of carrying a functioning pacemaker was itself associated with a reduced risk of recurrent syncope (odds ratio 0.16, 95% CI 0.06–0.40; *p* = 0.0001), which the authors attributed to a strong expectation effect [64]. The parallel with the current CNA literature is uncomfortable—the same population, the same outcome measure, the same open-label series reporting 80–90% success, the same absence of blinding—and it establishes that in this condition expectation alone can generate effect sizes of the magnitude now attributed to ablation. It does not follow that CNA will share the fate of pacing: pacing counteracts the consequence of the reflex and leaves no lasting autonomic signature, whereas CNA modifies the reflex itself and leaves durable, objectively measurable evidence of having done so. The precedent is an argument for completing sham-controlled trials, not for presuming their result.

That such trials remain incomplete reflects obstacles that are substantive rather than logistical. First, the control intervention is invasive: a credible sham requires vascular access, and plausibly transseptal puncture and catheter manipulation, in young patients with structurally normal hearts who are not at risk of dying from the condition being treated—an ethical threshold far above that of a placebo tablet. Second, the therapeutic target is a regulatory system with marked spontaneous variability: vagal tone fluctuates with sleep, training, hydration, emotional state, and season, so both substrate and outcome drift over follow-up independently of any intervention. Third, and compounding the other two, CNA has no binary procedural endpoint (Section 6): without a criterion equivalent to entrance block, success is inferred from surrogates that are themselves operator- and sedation-dependent, so a blinded trial cannot confirm that the active arm received an adequate intervention, and a neutral result becomes uninterpretable—indistinguishable from failure to denervate.

These constraints explain the design of the trials now under way: GENTLE-PACE and CARDIOBOOST both recruit patients who already carry an implanted device, permitting genuine blinding without exposing anyone to a purely sham ablation [59,60]. Their results should be regarded as a prerequisite for any upgrade of CNA from a reasonable option in selected patients to a guideline-recommended therapy.

### 7.6. Critical Appraisal of the Overall Level of Evidence

The evidence base remains dominated by observational data: most of the studies summarized in Table 4 are single-centre series, registries, or observational cohorts, and only three randomized trials have been published [8,16,34]. More consequential than their modest size is what they tested. ROMAN is the only randomized trial providing clinical outcome data against a non-ablation comparator [8]; ROMAN2 assessed an acute denervation endpoint rather than a clinical one [16]; and Tu et al. compared two ablation strategies against each other rather than CNA against an alternative therapy [34]. No completed randomized trial has therefore compared CNA with pacing, with pharmacological therapy, or with sham using a clinical primary endpoint. The efficacy of CNA relative to its alternatives has never been tested under randomization, and that gap is more fundamental than the modest sample sizes.

Three forms of bias plausibly inflate the apparent effect. Selection bias is probable, since most series originate from a small number of expert centres and may enrich for a well-defined cardioinhibitory phenotype and favourable anatomy (Section 7.7). Publication and reporting bias cannot be excluded, particularly for single-arm series reporting uniformly high success. Ascertainment bias is perhaps the least appreciated: follow-up commonly relies on symptom recall or on non-blinded repeat tilt testing rather than on continuous rhythm monitoring, so the outcome is measured considerably less rigorously than the intervention is delivered—an asymmetry that matters particularly in a condition whose defining event is intermittent, unwitnessed, and self-reported.

The certainty of the evidence supporting the efficacy of CNA should therefore be regarded as low to moderate rather than high. The limiting factor is not the size of the reported effects, which is large and consistent across study designs, but the fact that these effects arise from the designs least able to exclude the biases above; large and consistent effects would ordinarily increase confidence in a causal interpretation, and here they cannot. The mechanistic coherence set out in Section 3 and Section 8 is real and is captured by none of these considerations—but coherence is not efficacy, and that is why the question will not be settled by mechanism alone.

### 7.7. Generalizability Beyond Expert Centers: Learning Curve and Operator Dependency

How far the evidence in this review generalizes beyond a small number of high-volume centres is largely unaddressed. The 2024 EHRA survey of 202 physicians from 40 countries offers the most direct view of real-world practice: only half of respondents had performed a single CNA in the preceding 12 months, and among those who had not adopted the technique, lack of experience was the most frequent barrier (29%), ahead of concerns about the risk–benefit profile (19%), the absence of guideline coverage (18%), and long-term effects (17%) [65]. What limits dissemination, in other words, is not scepticism about whether CNA works, but uncertainty about how to do it.

Among centres that have adopted it, the same survey describes a field that has adopted the procedure without adopting a procedure. Biatrial ablation was first-line for 71%, while 16% reserved the left atrium for right-atrial non-responders; GP localization rested predominantly on anatomical landmarks (90%) and electrogram analysis (59%), with high-frequency stimulation (17%), spectral analysis (10%), and pre-procedural imaging (20%) used far less consistently; and procedural success was most often judged by a simple heart-rate increase rather than by a validated denervation marker [65]. The absence of a standardized endpoint (Section 6) is therefore not merely a methodological problem for trials: it is what everyday practice looks like, and it is the most plausible reason why results obtained under rigorously defined protocols may not reproduce across a broader operator base.

The cost of that variability is hard to quantify. The survey’s reassuring complication estimates (<1% for 71% of respondents) are physician-recalled rather than adjudicated, and systematically collected registries report higher rates than the pioneering single-centre series (Section 8.1) [10,11]. Direct evidence is sparse and, so far, neutral: PIRECNA is the only study to have tested operator dependency prospectively, and neither operator experience (hazard ratio 0.52, 95% CI 0.19–1.41) nor use of extracardiac vagal stimulation (hazard ratio 0.54, 95% CI 0.2–1.41) was significantly associated with outcome—although, with 130 patients and 17 events, the registry could not have detected a moderate effect, and both point estimates favoured the more experienced operators and the more rigorously monitored strategy [28]. The 2024 EHRA/HRS/APHRS/LAHRS statement accordingly recommends that CNA be regarded as investigational outside well-defined indications until reproducible training standards and outcome benchmarks are established [3]. Establishing them will require multicentre studies powered to treat operator experience and procedural volume as pre-specified covariates, not merely to report them.

## 8. Safety, Adverse Events, and Long-Term Durability

The safety of CNA should be considered at two levels: the acute procedural safety of right- and/or left-atrial catheter ablation, and the long-term physiological consequences of intentionally shifting cardiac autonomic balance in patients who are often young and structurally healthy. Unlike ablation of a re-entrant tachycardia, CNA modifies a regulatory system. The relevant safety question is therefore not only whether the procedure can be performed with few acute complications, but also whether the extent of vagal withdrawal is appropriate, durable, and free from clinically meaningful adverse autonomic effects.

### 8.1. Procedural Safety and Complications

Available randomized and observational data suggest that CNA can be performed with a low acute major complication rate in experienced centers. The potential complications overlap with those of other atrial ablation procedures: vascular access complications, transseptal puncture complications, pericardial effusion or tamponade, thromboembolism, pericarditis, phrenic nerve injury, coronary or sinus-node-artery injury, sinus node or AV node damage, and pulmonary vein stenosis [8,51,52,53].

Real-world data, however, are more cautionary. In the United States Multicenter CNA Registry, complications occurred in 4.7% of procedures, with a 1.4% major adverse event rate including hemopericardium, diaphragmatic paralysis, respiratory failure, and death [11]. The Spanish multicenter series reported a 6.5% complication rate across 22 hospitals [10]. These findings suggest that safety is influenced by center experience, patient selection, imaging integration, and procedural endpoint rigor.

Because CNA is performed for a condition that is usually not life-threatening, the acceptable threshold for harm must remain low. Careful selection, avoidance of excessive ablation, awareness of adjacent structures, phrenic nerve localization, conservative lesion delivery near nodal tissues, and objective procedural endpoints should be regarded as safety requirements rather than optional refinements.

Beyond conventional ablation complications, preclinical data raise the possibility that extensive vagal denervation may have proarrhythmic consequences in vulnerable settings. In a chronic porcine CNA model, denervation persisted at 6 weeks but was associated with autonomic dysreflexia, exaggerated response to sympathetic stimulation, corrected QT interval (QTc) prolongation, and earlier ventricular tachyarrhythmias during acute coronary occlusion [20,66]. These findings should not be directly extrapolated to young patients without structural disease, and no clinical signal of increased sudden cardiac death has emerged, but they are a useful warning: vagal tone is not merely a mediator of syncope; it is also cardioprotective. CNA should therefore be conceived as targeted neuromodulation, not autonomic ablation at any cost.

### 8.2. Inappropriate Sinus Tachycardia

Sinus-rate acceleration is an expected physiological effect of CNA and one of its most visible markers of parasympathetic attenuation. When excessive, persistent, or symptomatic, however, this shift may manifest as inappropriate sinus tachycardia (IST), with palpitations, fatigue, dyspnea, exercise intolerance, or anxiety-like symptoms.

IST reflects sympathovagal imbalance after preferential vagal attenuation and usually improves over months through partial reinnervation or autonomic adaptation. A clinically relevant minority requires treatment: reported estimates suggest post-CNA IST in approximately 7–8%, and in one medium-term cohort, symptomatic sinus acceleration occurred in about one quarter of patients, with chronic beta-blocker or ivabradine therapy required in 7% [67].

Management should be stepwise: exclude secondary causes, reassure when symptoms are mild and early, optimize hydration and graded exercise, and use ivabradine or low-dose beta-blockers when necessary. Sinus-node modification should be exceptional because it may recreate the original bradycardia problem or lead to pacemaker implantation [68]. Clinically significant IST is also a procedural signal: denervation should be sufficient to prevent cardioinhibition but not so extensive that physiological autonomic balance is unnecessarily disrupted.

### 8.3. Reinnervation and Late Recurrence

The durability of CNA depends on whether clinically meaningful parasympathetic attenuation persists, and the principal threat to that durability is reinnervation. Its likelihood is governed by a single distinction, drawn from other settings of cardiac autonomic injury: damage to the neuronal cell body is effectively permanent, whereas injury confined to axons is followed by regeneration and collateral sprouting, so that spared neurons progressively reinnervate the myocytes of ablated ones and function returns, albeit in a patchy pattern [3,13]. This is precisely why CNA targets the ganglionated plexus neurons themselves rather than the postganglionic fibres, and why the completeness of the initial lesion is decisive. Comparable reinnervation is well documented after cardiac transplantation, most likely in the sinoatrial nodal region of the allograft, and after stellate ganglion surgery [3].

In humans, the time course is inferred almost entirely from serial heart-rate and HRV measurements rather than from imaging or histology. The most informative dataset is a prospective study of 83 patients with denervation confirmed at the index procedure (by ECVS in 78%), in whom 24 h Holter HRV was compared before ablation and at 1 and 2 years: every time- and frequency-domain parameter remained significantly reduced at 2 years (by 33–85% and 71–82%, respectively; *p* < 0.001), with no significant change between years 1 and 2 [69]. Whatever reinnervation occurs, therefore, appears largely complete within the first year and then stabilizes. Where later recovery of heart rate and HRV toward baseline has been described, it has been greater in patients with recurrence or a positive follow-up tilt test, but the data remain insufficient to establish that the degree of reinnervation predicts recurrence, and its extent varies with both procedural factors (lesion technology and extent, accuracy of GP targeting) and patient factors (atrial wall thickness, epicardial fat, individual regenerative capacity) [13]. Critically, no CNA series has reported outcomes beyond five years, and the longest available follow-up—approximately 45 months—falls short of the horizon over which slow axonal regrowth might become clinically apparent [7,13].

A consistent and clinically important observation is the dissociation between autonomic markers and symptoms: HRV indices may partially recover while patients remain syncope-free, which suggests that only partial denervation is needed to interrupt the pathological reflex, or that CNA produces more durable neural remodelling than simple axonal injury would imply. The corollary is that reinnervation on a monitor is not the same as clinical failure, and the two should not be equated. Reassuringly, outcome data support durability: the Pachon group reported over 93% freedom from syncope at a mean follow-up beyond 45 months in ECVS-confirmed patients [7], and in a 115-patient cohort 83% remained syncope-free over a median of 28 months, with markedly reduced syncope burden among the few who recurred, repeat CNA in 3%, and pacemaker implantation in 4% [49].

Clinically, late recurrence after an asymptomatic interval should nonetheless raise the possibility of reinnervation, particularly when accompanied by a fall in resting heart rate toward pre-ablation values or renewed atropine responsiveness. This sequence has been documented directly: in a patient with initially effective CNA, implantable loop recorder trends showed heart rate and HRV drifting back to preprocedural values until syncope recurred—a trajectory attributed to suboptimal initial denervation rather than to inevitable reinnervation, and a reminder that recovery is not always unwanted, since attenuation of post-procedural sinus tachycardia may itself be desirable [70].

No single method reliably distinguishes benign reinnervation from impending clinical failure. Serial resting and ambulatory heart rate and HRV are the most accessible tools and were sufficient to demonstrate durable denervation at two years [69], but HRV is relatively insensitive to reinnervation and is confounded by age, conditioning, medication, and intercurrent illness; repeat atropine testing is more reproducible yet cannot be performed often [13]; and ECVS, though the most specific, is invasive and confined to the periprocedural setting. A pragmatic follow-up protocol therefore combines serial heart-rate and HRV assessment with implantable loop recorder monitoring in patients with traumatic or high-risk presentations—events while driving, occupational hazard, or diagnostic uncertainty—reserving repeat head-up tilt testing for selected cases, where it informs but does not substitute for documented spontaneous recurrence [69].

### 8.4. Quality of Life

Quality of life is a central outcome because reflex syncope rarely threatens survival but can profoundly limit independence, driving, work, sport, social activity, and psychological well-being. For many patients, disease burden is driven as much by unpredictability and fear of injury as by the absolute number of syncopal episodes.

Available data suggest meaningful quality-of-life improvement after CNA in selected patients with predominantly cardioinhibitory VVS. In ROMAN, the University of Calgary Impact of Syncope score improved by approximately 67% in the CNA group and did not improve in controls [8]. A prospective cohort using the 36-Item Short Form Health Survey (SF-36) and the EuroQol 5-Dimension/visual analogue scale (EQ-5D/EQ-VAS) reported freedom from syncope at 12 months, negative repeat HUTT in 85%, and significant improvement in both instruments [71]. Ongoing trials appropriately include validated quality-of-life instruments as secondary outcomes.

## 9. Ongoing Trials, Knowledge Gaps, and the Road Ahead

### 9.1. Trials in Progress

The decisive development in the field is the transition from observational experience to randomized, controlled, and—where feasible—sham-blinded trials. A review of registered studies identified twelve ongoing randomized trials across the spectrum of vagally mediated bradyarrhythmias, with planned enrolment exceeding 1400 patients [59]. The designs, populations, and primary endpoints of those most likely to shape practice are summarized in Table 6; they can be grouped by the specific question each is built to answer.

Two trials confront the placebo-response critique directly, and are for that reason the most consequential. GENTLE-PACE (NCT05896592) randomizes patients with an already implanted device to CNA, continued pacing, or a double-blind sham intervention, so that blinding is achieved without exposing anyone to a purely sham ablation [60]; CARDIOBOOST (NCT06288633) provides a second sham-controlled framework spanning both sinus node dysfunction and AV block phenotypes [59]. Together they will test whether the physiological signature of CNA translates into a blinded, patient-relevant clinical benefit.

A second group positions CNA against its real alternatives. PANACEA (NCT05855603) addresses the central equipoise—whether pacing or CNA should come first in a patient older than 40 years with cardioinhibitory reflex syncope who already meets pacing criteria—by randomizing to one therapy or the other [59]. CAMPAIGN (NCT05803148) instead compares CNA with midodrine in tilt-positive VVS, testing it against pharmacological rather than device therapy [72].

A third group extends randomized evaluation to the phenotypes least represented in the current literature: SAN.OK (NCT05196126) in functional sinus node dysfunction [73], and TELE-SPACER (NCT05774262) in functional high-grade AV block, the latter using implantable loop recorder documentation for endpoint adjudication [74]. Collectively, whether these trials convert a consistent open-label signal into blinded, phenotype-specific, guideline-grade evidence will determine whether CNA becomes an established therapy rather than a promising expert-centre practice.

### 9.2. Unsolved Questions and Methodological Gaps

Current evidence for CNA is characterized by a consistent medium-term efficacy signal and acceptable safety in experienced hands, but the field now needs to move from expert-center innovation to reproducible clinical pathway. The decisive questions are no longer whether CNA is anatomically plausible or technically feasible, but whether its clinical benefit is placebo-adjusted, its candidates are reliably identifiable, its procedural endpoint is measurable, its denervation strategy is proportionate, and its long-term autonomic effects are safe and durable (Table 7).

The first priority is a blinded estimate of efficacy. VVS is prone to spontaneous remission, regression to the mean, and expectation effects, so open-label studies cannot fully separate ablation-specific benefit from the non-specific effects of procedural attention [75]. ROMAN provides an important randomized efficacy signal, but its control arm did not include sham intervention or active pharmacological therapy [8]. GENTLE-PACE and CARDIOBOOST are therefore pivotal because they will test whether the physiological signature of CNA translates into a blinded, patient-relevant clinical benefit.

The second priority is harmonized phenotyping. Reflex syncope is not a sufficient indication; the relevant substrate is dominant cardioinhibition with preserved intrinsic SA or AV nodal reserve and limited competing vasodepressor burden. Future studies should standardize how HUTT, carotid sinus massage, ILR documentation, atropine response, baseline conduction testing, and blood-pressure phenotype are combined. Cardiac deceleration capacity is particularly attractive because it quantifies vagal predominance on a continuous scale, but it requires prospective validation as an enrollment criterion rather than as a post hoc marker of response [18].

The third priority is an outcome-linked procedural endpoint. ECVS is the most direct available test of functional denervation, but protocols, thresholds, and availability remain heterogeneous [12,49]. Heart-rate acceleration, attenuation of the atropine response, abolition of HFS-evoked vagal responses, PR shortening, and Wenckebach-point normalization are useful but not interchangeable. The field should move toward phenotype-specific endpoint sets: SA-node denervation for sinus arrest or functional SND, AV-node denervation for functional AV block, and integrated autonomic endpoints for mixed phenotypes.

The fourth priority is proportional ablation. Comprehensive biatrial denervation may maximize the acute autonomic effect, but may also increase lesion burden and the risk of excessive denervation. Conversely, overly conservative ablation may leave residual nodal vagal input or incomplete clinical effect. The ELEGANCE tailored strategy and the randomized left-atrial versus biatrial trial support selective, phenotype-guided ablation as a way to reduce procedural burden without compromising medium-term efficacy [17,34]. Longer follow-up is required to determine whether this advantage persists as vagal reinnervation occurs.

The fifth priority is long-term surveillance of both efficacy and safety. Reinnervation may be biological, partial, and clinically silent, or it may explain late recurrence; current studies have not consistently linked serial autonomic testing to rhythm-documented outcomes. Follow-up should therefore combine symptoms, injury burden, quality of life, ambulatory ECG or ILR data, HRV or deceleration capacity, atropine testing, and, where available, ECVS or repeat HUTT. The same framework should capture excessive denervation phenotypes, including persistent inappropriate sinus tachycardia, marked HRV suppression, QT changes, atrial arrhythmias, and any signal suggesting loss of the cardioprotective effects of vagal tone [66].

Finally, CNA must be positioned against existing therapies. The key unresolved scenario is the 40–60-year-old patient with documented cardioinhibitory reflex syncope who meets conventional pacing criteria but is otherwise structurally normal and device-averse. Pacing is guideline-supported and reliable for preventing asystolic pauses; CNA is mechanism-directed and avoids lifelong hardware but remains less proven. PANACEA directly addresses this equipoise by randomizing patients older than 40 years with an accepted pacing indication to CNA or permanent pacing [59]. Until these data mature, shared decision-making should weigh age, spontaneous rhythm documentation, vasodepressor burden, atropine response, comorbidity, occupational risk, patient preference, and tolerance for uncertainty.

Underlying all of these is the challenge of implementation: because outcomes in broader practice may fall short of expert-centre series (Section 7.7), translating CNA into routine care will depend less on any single technical refinement than on common data elements, standardized training and adverse-event definitions, and prospective registries capturing patient-reported as well as rhythm outcomes.

### 9.3. Future Directions

Beyond the randomized trials summarized in Section 9.1, several technical and conceptual developments are likely to shape the next phase of CNA. A recent controlled observational study evaluated biatrial CNA guided by ultra-high-density electroanatomical mapping with automated fragmented-electrogram detection and robotic magnetic navigation in 12 patients with drug-refractory, vagally mediated bradyarrhythmias, compared with 10 medically managed controls; the procedure was feasible and safe, with minimal fluoroscopy exposure, a substantial increase in resting sinus rate, and fewer recurrences and pacemaker implantations than in the control group over approximately 12 months of follow-up [76]. Although based on a small, retrospective cohort and therefore hypothesis-generating rather than practice-changing, this experience illustrates how automated, algorithm-guided electrogram analysis and remote navigation could reduce operator dependency and improve the reproducibility concerns discussed in Section 7.7.

A second area of active development is procedural endpoint standardization through provocative testing. The Vagal AF Induction Test (VAFIT), which pairs extracardiac vagal stimulation with measurement of the effective atrial refractory period to assess inducibility of atrial fibrillation before and after ablation, was recently evaluated in 142 patients undergoing combined pulmonary vein isolation and CNA for atrial fibrillation. A VAFIT-negative result at the end of the procedure was independently associated with a markedly lower rate of arrhythmia recurrence (hazard ratio 4.56 for VAFIT-positive versus VAFIT-negative patients) [77]. Although developed and validated in the context of vagally mediated atrial fibrillation rather than reflex syncope, VAFIT exemplifies the kind of objective, physiology-based intraprocedural endpoint called for in Section 6, and its underlying ECVS-based framework is directly transferable to endpoint research in cardioinhibitory VVS and functional bradyarrhythmias.

A third emerging direction concerns the energy source used for denervation. Preclinical and early clinical work has explored epicardial pulsed-field ablation (PFA) as a means of selectively electroporating ganglionated plexi while sparing the underlying myocardium, based on the differential electroporation thresholds of neuronal and myocardial cell membranes. Because PFA ablates through a non-thermal mechanism, it may avoid the thermally mediated release of nerve growth factors thought to promote axonal regrowth after radiofrequency ablation, potentially reducing the risk of reinnervation discussed in Section 8.3; however, this hypothesis has not yet been tested in the context of reflex syncope or functional bradyarrhythmias, and current PFA experience with epicardial ganglionated plexus ablation has focused on atrial fibrillation and post-operative arrhythmia prevention rather than on cardioinhibitory VVS [78]. Whether an epicardial, PFA-based approach could eventually be adapted to CNA for reflex syncope remains speculative and would require dedicated feasibility and safety studies given the very different access route (epicardial versus endocardial) currently used in this field.

A unifying feature of these developments is that they target reproducibility rather than efficacy: automated mapping and remote navigation aim to reduce operator dependency, objective provocative endpoints to standardize the definition of success, and non-thermal energy to make denervation more durable. Each addresses a specific weakness identified earlier in this review, and each will require the same phenotypic rigour and controlled comparison demanded of the therapy itself before it can be considered established.

### 9.4. Limitations of This Review

This review has several limitations inherent to its narrative design. First, as detailed in Section 2, reference selection followed an iterative, section-by-section process rather than a single systematic search executed against a pre-registered protocol, and no formal risk-of-bias assessment or quantitative synthesis was performed. This is appropriate for a state-of-the-art review intended to provide a clinically oriented, mechanistically grounded synthesis, but—despite cross-checking against the major consensus documents [3,13]—it carries a risk of selection bias that a systematic review would more directly mitigate.

Second, the limitations of this synthesis are inseparable from those of the evidence it summarizes. As appraised in Section 7.6, that evidence is dominated by observational, often single-centre data with heterogeneous methodology and only three published randomized trials; the frequently cited 80–94% freedom-from-syncope range should therefore be read as an approximate, evolving estimate rather than a definitive figure.

Third, the field is moving quickly. Several studies and consensus documents cited here appeared within the past one to two years, and the forthcoming sham-controlled trials (Section 9.1) may materially revise the evidentiary landscape within a short time frame; the quantitative estimates given here are a snapshot at the time of writing.

Finally, as with any narrative review written by investigators active in the field, the authors’ own experience—including multimodal GP localization and the management of late functional AV block after AVNRT ablation (Section 5.3 and Section 4.4)—may have shaped the emphasis placed on particular techniques, despite a deliberate effort to present competing approaches even-handedly and to flag genuine uncertainty throughout.

## 10. Conclusions

CNA has evolved from a single group’s proof of concept into a procedure evaluated by international societies, randomized trials, registries, and meta-analyses. Its efficacy signal is consistent—approximately 80–94% freedom from recurrent syncope in carefully selected patients—and its mechanism is anatomically persuasive: endocardial radiofrequency reaches atrial GP, attenuates postganglionic parasympathetic transmission to the SA and AV nodes, and produces measurable changes in heart rate, AV nodal conduction, atropine response, and HRV. In young, structurally normal patients with a documented cardioinhibitory phenotype, CNA offers the most plausible mechanism-directed alternative to lifelong device therapy.

The central evidentiary gap is the absence of completed sham-controlled trials. This is not a methodological detail. VVS is placebo-responsive and often remitting, so the specific contribution of ablation must be distinguished from natural history, regression to the mean, and the non-specific effects of an invasive procedure. The physiological signature of CNA makes spontaneous remission an unlikely sole explanation for the published outcomes, but guideline endorsement requires proof, not plausibility. GENTLE-PACE and CARDIOBOOST will therefore be decisive. Positive results would support integration of CNA into formal treatment pathways; neutral or negative results would require the field to recalibrate its claims.

Until those data are available, success in clinical practice depends on two inseparable conditions: selecting the right patient and achieving the right autonomic effect. The cardioinhibitory substrate must be documented, not assumed. The procedural endpoint must be objective, ideally physiological, and linked to the target phenotype. ECVS remains the most direct intraprocedural assessment of functional denervation, although broader standardization is needed. The procedural aim should be calibrated denervation: enough to prevent pathological cardioinhibition, not so much that inappropriate sinus tachycardia or other adverse autonomic consequences become likely. Two decades after its first description, CNA has matured from an anatomically grounded hypothesis into an evidence-supported, mechanism-directed intervention whose definitive place in clinical practice will be determined by the ongoing randomized trials outlined above.

## Figures and Tables

**Table 1:** Nomenclature of the principal atrial ganglionated plexi relevant to cardioneuroablation: anatomical, electroanatomical, and paraseptal systems.

Anatomical/Legacy Nomenclature	Contemporary Electroanatomical Nomenclature	Paraseptal/Functional Nomenclature	Refined Anatomical Location
Superior right atrial GP	Right superior GP (RSGP)	SPSGP	High paraseptal/posterosuperior RA at the SVC–RA junction, adjacent to the right superior PV–SVC interatrial region
Posterior right atrial GP	Right inferior GP (RIGP)	—	Inferoposterior right atrial/interatrial region within the right inferior fat pad (pyramidal space), adjacent to the CS ostium, septal IVC–RA junction, and anterior-inferoseptal aspect of the right inferior PV
Superior left atrial GP	Left superior GP (LSGP)	—	Posterosuperior LA, adjacent to the left superior PV–LA junction
Posteromedial left atrial GP	Posteromedial left GP (PMLGP)	IPSGP	Inferoposterior paraseptal LA at the interface between the posterior LA wall and CS ostium, adjacent to the posterior interatrial groove; functionally linked to AV nodal modulation
Posterolateral left atrial GP	Left inferior GP (LIGP)	—	Posterolateral–inferior LA, posterior to the left inferior PV and extending toward the lateral mitral isthmus
Marshall tract/ligament of Marshall GP	Marshall tract GP (MTGP)	—	Epicardial autonomic region of the Marshall complex along the left lateral ridge, between the LAA and left PVs; procedurally targeted at the left PV carina or adjacent ridge
Aorto-caval GP	Ao-SVC GP/SVC-Ao GP	—	High right superior epicardial autonomic region between the SVC and ascending aorta, along the posteromedial SVC wall and anterolateral ascending aortic wall, inferior to the right pulmonary artery

Ao, aorta; Ao-SVC GP, aorta–superior vena cava ganglionated plexus; AV, atrioventricular; CS, coronary sinus; GP, ganglionated plexus; IPSGP, inferior paraseptal ganglionated plexus; IVC, inferior vena cava; LA, left atrium; LAA, left atrial appendage; LIGP, left inferior ganglionated plexus; LSGP, left superior ganglionated plexus; MTGP, Marshall tract ganglionated plexus; PMLGP, posteromedial left ganglionated plexus; PV, pulmonary vein; RA, right atrium; RIGP, right inferior ganglionated plexus; RSGP, right superior ganglionated plexus; SPSGP, superior paraseptal ganglionated plexus; SVC, superior vena cava; SVC-Ao GP, superior vena cava–aorta ganglionated plexus.

**Table 2:** Comparative overview of the principal ganglionated plexus localization strategies used in cardioneuroablation.

Technique and Principle	Main Advantages	Main Limitations	Practical Considerations
**Anatomical targeting**: RF delivery to predefined GP regions using anatomical landmarks and electroanatomic reconstruction.	Available in any electrophysiology laboratory; rapid and reproducible; no dedicated technology is required.	Anatomical variability; no site-specific functional confirmation; risk of missed targets or unnecessary lesions.	Use as the basic scaffold; prioritize phenotype-relevant GP, account for wall thickness and vulnerable structures, and extend only if the intended endpoint is not achieved [3,17,32].
**High-frequency stimulation (HFS)**: bradycardia, asystole, or AV block during stimulation identifies functionally active GP.	Patient-specific functional confirmation; useful when anatomical and electrogram findings are discordant.	Protocol variability; sedation- and depth-dependent responses; AF induction; possible false-negative results at deep targets.	Map before ablation and atropine; typical trains use 10–20 Hz for 2–5 s; synchronized stimulation may reduce AF induction [35,36,37,38].
**Fractionated electrogram (fEGM) mapping**: multicomponent near-field signals identify presumed GP-containing myocardium.	Compatible with high-density mapping; no autonomic provocation; systematic target annotation.	Low specificity; fibrosis, scar, anisotropy, and far-field signals also fractionate; results are settings-dependent.	Report catheter, filters, and criteria (e.g., 200–500 Hz and deflection count); combine with anatomical and/or functional mapping [36,39,40].
**Spectral/peak-frequency mapping, including omnipolar technology near field (OTNF)**: detects high-frequency near-field components over GP-associated fibrillar tissue.	Automated or semi-automated; less subjective; complementary to fractionation mapping.	Limited validation; platform-dependent thresholds; spatial discordance with fEGM maps.	Use as an adjunct, not a stand-alone target or endpoint; report the threshold used (approximately 450–600 Hz) [41,42,43].
**Computed tomography (CT) fat-pad segmentation**: epicardial fat (−190 to −30 HU) is fused with the electroanatomic map.	Patient-specific 3-dimensional anatomy; defines fat-pad extent, wall thickness, and adjacent structures.	Fat is a surrogate; registration error and imperfect functional correlation; additional processing, radiation, and contrast.	Verify registration; combine with electrogram or functional mapping; useful for multimodal planning [4,43,44].
**Intracardiac echocardiography (ICE)**: real-time visualization of right paraseptal fat pads and the phrenic nerve.	No pre-procedural CT; rapid reconstruction; supports right-sided targeting and low-fluoroscopy workflows.	Mainly right-sided evidence; operator-dependent; additional access and cost; limited comparative data.	Reconstruct the SVC-RA fat pad, pyramidal space, and phrenic nerve before RF delivery [45].

AF, atrial fibrillation; AV, atrioventricular; CT, computed tomography; fEGM, fractionated electrogram; GP, ganglionated plexus; HFS, high-frequency stimulation; HU, Hounsfield unit; ICE, intracardiac echocardiography; OTNF, omnipolar technology near field; RF, radiofrequency; SVC-RA, superior vena cava-right atrium.

**Table 3:** Procedural endpoints used in cardioneuroablation: advantages and limitations.

Endpoint	Advantage	Limitation
Resting heart-rate increase (≥20–25% from baseline)	Simple, objective, widely reported; correlates with freedom from syncope and negative tilt at follow-up	Non-specific; depends on baseline rate or sedation depth
Abolition of the atropine response	Denervation is considered adequate when the heart-rate response to atropine is <10%	No universally agreed threshold (10–25% acceleration); delayed assessment; once performed, the test confounds the procedure and cannot be repeated
Abolition of HFS-evoked vagal response	Physiological, site-specific confirmation of functional denervation	Technically demanding; may induce AF; sedation-dependent; atropine interferes
Extracardiac vagal stimulation (ECVS) response	Most direct test of global vagal denervation; allows stepwise monitoring in experienced hands	Specialized equipment and expertise; limited availability
Antegrade Wenckebach-point normalization	Phenotype-specific endpoint for AV nodal denervation; clinically intuitive in functional AV block	Only relevant for AV block phenotype; no agreed threshold

ECVS, advocated by the Pachon group as the most direct intraprocedural test of functional denervation, stimulates the cervical vagus nerve while the cardiac response is monitored. Abolition of the induced cardioinhibitory response indicates that the efferent vagal pathway to the nodal targets has been effectively interrupted. In a prospective controlled study of 62 patients, atrial fibrillation nest (AF-Nest) ablation guided by ECVS abolished the ECVS-induced response in all treated patients, whereas conventional ablation in controls produced no significant denervation [49]. The principal advantages of ECVS are physiological specificity and stepwise monitoring; the principal limitation is limited availability of equipment and experience.

**Table 4:** Key clinical studies of cardioneuroablation for reflex syncope and functional bradyarrhythmias.

Study (Year)	Design	N	Population	CNA Approach	Follow-Up	Recurrence/Major Adverse Events
Pachon (2005) [6]	Prospective, single-arm	21	VVS, functional AV block, SND	Spectral mapping, biatrial	~9 mo	Symptom relief in all 21; no complications reported
Pachon (2011) [7]	Observational	43	Refractory neurally mediated syncope	Spectral mapping, biatrial	~45 mo	3 recurrences (7%); atropine negative in 77%; no major adverse events reported
ROMAN (2023) [8]	RCT, open-label	48	Cardioinhibitory/mixed VVS	Biatrial	2 yr	Recurrence 8% CNA vs. 54% control (*p* = 0.0004); no major adverse events
ROMAN2 (2024) [16]	RCT, acute endpoint	40	Reflex asystolic syncope	RA-first vs. LA-first	Acute	Acute endpoint only: complete denervation 35% RA-first vs. 80% LA-first (*p* = 0.01)
Tu et al. (2025) [34]	RCT, non-inferiority	80	VVS	LA-only vs. biatrial	12 mo	Syncope-free 87.5% LA-only vs. 90% biatrial (non-inferior; *p* = 0.723)
ELEGANCE tailored (2026) [17]	Prospective multicentre	123	Reflex syncope/functional bradycardia	Standard vs. phenotype-guided	15.9 mo	Equivalent syncope-free survival (log-rank *p* = 0.96); acute success 93% vs. 90%
US Multicenter Registry (2025) [11]	Prospective multicentre	205	VVS, SND, AV block	Anatomical ± HFS; biatrial 77%	14 mo	78% syncope-free; complications 4.7%, major adverse events 1.4% (incl. one death)
PIRECNA (2024) [28]	International multicentre registry (20 centres)	130	Symptomatic paroxysmal or persistent vagally induced supra-Hisian AV block	Electroanatomical-guided; ECVS in 56%	300 d (median)	Acute success 96.2%; primary outcome 17/125 (14%); 77.3% free of events at 360 d; 4 required pacing; no major procedure-related complications
Barrio-López Spain (2024) [10]	Multicentre real-world	77	Consecutive VVS	Variable	12 mo	66.2% syncope-free; complications 6.5%
Vandenberk meta-analysis (2022) [2]	Meta-analysis	465	Mixed	Mixed	Mixed	Freedom from syncope 91.9% (95% CI 88.1–94.6%); RA-only inferior
Armani Prata meta-analysis (2025) [9]	Meta-analysis	1153	Mixed	Mixed	Mixed	Recurrence 5.94% (95% CI 3.37–9.01%); periprocedural complications 0.99%

AV, atrioventricular; CNA, cardioneuroablation; ECVS, extracardiac vagal stimulation; HFS, high-frequency stimulation; LA, left atrium; RA, right atrium; RCT, randomized controlled trial; RF, radiofrequency; SND, sinus node dysfunction; VVS, vasovagal syncope.

**Table 5:** Comparison between cardioneuroablation and permanent pacing in cardioinhibitory reflex syncope.

Domain	Cardioneuroablation (CNA)	Permanent Pacing
Mechanism	Attenuates postganglionic parasympathetic input to the SA/AV nodes at its source; addresses the autonomic trigger	Prevents asystolic pauses electrically; does not modify the autonomic reflex or the vasodepressor component
Guideline status	Not yet guideline-endorsed; positioned by the 2024 EHRA/HRS/APHRS/LAHRS statement as a reasonable option in selected patients [3]	Guideline-endorsed (2021 ESC) in patients > 40 years with severe, unpredictable, recurrent cardioinhibitory reflex syncope and documented spontaneous asystole [5]
Advantages	Device-free; potentially addresses the underlying mechanism; avoids lifelong hardware; attractive for younger, active, device-averse patients. Reported freedom from syncope ranges from 78% in the US multicenter registry to 88% in ELEGANCE and approximately 92% in meta-analyses, with 97% remaining pacemaker-free in the US registry, and quality of life improves [2,8,11,17,22]	Long track record; reliable prevention of asystolic pauses; standardized implantation, programming, and follow-up
Limitations	No completed sham-controlled efficacy trial; heterogeneous indications, workflow, and endpoints across studies; no universally validated procedural endpoint; durability may be limited by vagal reinnervation (Section 8.3); does not address the vasodepressor component [3,10,39]	Does not treat vasodepressor component; lifelong device dependency; cumulative lead/generator/infection risk over decades [5]
Risks/complications	Vascular access, pericardial effusion/tamponade, phrenic nerve or sinus-node-artery injury, inappropriate sinus tachycardia (~7–8%); complications in 4.7% and major adverse events ~1.0–1.4% in real-world registries, with lower rates in experienced centres [10,11]	Lead fracture/dislodgement, pocket infection or erosion, pneumothorax, tricuspid regurgitation, device-related endocarditis, need for generator/lead replacement over time
Strength of evidence	Moderate but still evolving: three small RCTs (ROMAN, ROMAN2, Tu et al.) plus observational registries and meta-analyses; only one non-randomized comparison directly against pacing; sham-controlled multicentre trials still pending [8,9,16,34,55,59,60]	Robust RCT evidence for closed-loop and dual-chamber pacing in reflex syncope (e.g., SPAIN, BioSync CLS) [56,57]; decades of guideline endorsement, large registries, and systematic reviews of complications
Ideal patient profile	Younger patients (particularly <60 years), structurally normal heart, clearly documented cardioinhibitory phenotype, preserved atropine response, strong preference to avoid a device; less certain in carotid sinus syndrome or a dominant vasodepressor phenotype [3,13,22,23]	Older patients (generally >40–60 years), intrinsic or irreversible conduction disease, comorbidities favoring a simpler device-based solution, or CNA failure/contraindication

AV, atrioventricular; CNA, cardioneuroablation; CLS, closed-loop stimulation; ELEGANCE, cardionEuroabLation: patiEnt selection, imaGe integrAtioN and outComEs study; ESC, European Society of Cardiology; RCT, randomized controlled trial; SA, sinoatrial.

**Table 6:** Ongoing randomized controlled trials of cardioneuroablation.

Trial (NCT)	Design	Population	N (Planned)	Primary Endpoint	Est. Completion
PANACEA (NCT05855603)	Multicenter, open-label RCT	Cardioinhibitory reflex syncope, age >40 y, accepted pacing indication	90	12-month syncope-free survival	Feb 2028
GENTLE-PACE (NCT05896592)	Multicenter, double-blind, sham-controlled RCT (CNA vs. continued pacing vs. sham)	Patients with an implanted pacemaker for symptomatic bradycardia	NR	Composite efficacy (syncope/presyncope/ILR-detected bradycardia) and composite safety	~2029
SAN.OK (NCT05196126)	Multicenter, single-blind RCT	Functional sinus node dysfunction	60	Freedom from bradycardia symptoms and pacemaker avoidance at 6 months	Unknown
TELE-SPACER (NCT05774262)	Multicenter, randomized, unblinded, proof-of-concept trial + registry	Functional AV block with accepted pacing indication	100 (trial) + up to 200 (registry)	Feasibility and non-inferiority of CNA-guided strategy vs. pacing	Dec 2026
CAMPAIGN (NCT05803148)	Multicenter, open-label RCT	VVS with positive tilt test	184	Syncope recurrence at 12 months	~2026
CARDIOBOOST (NCT06288633)	Multicenter, sham-controlled RCT	Bradyarrhythmia (sinus node and AV node dysfunction)	NR	NR	Feb 2027

AV, atrioventricular; CNA, cardioneuroablation; ILR, implantable loop recorder; NR, not reported in the public trial registry; RCT, randomized controlled trial; VVS, vasovagal syncope.

**Table 7:** Key unresolved questions and future research priorities in cardioneuroablation.

Domain	Why It Matters	Research Priority
**Placebo-adjusted efficacy and sham-controlled evidence**	Open-label studies cannot fully separate the specific treatment effect of CNA from spontaneous remission, regression to the mean, and expectation effects.	Complete adequately powered randomized trials, including blinded or sham-controlled designs when feasible.
**Patient selection beyond “cardioinhibitory syncope”**	Cardioinhibition alone is insufficient; the ideal candidate should have a dominant vagal mechanism, preserved intrinsic nodal reserve, and limited competing causes of syncope.	Standardize phenotyping using clinical history, HUTT, ILR documentation, carotid sinus massage, atropine response, baseline conduction assessment, and exclusion of intrinsic disease.
**Quantifying the vasodepressor burden**	CNA targets the cardioinhibitory limb but does not directly treat vasodepression, which may explain persistent symptoms or recurrence despite effective denervation.	Incorporate blood-pressure phenotype, hypotensive susceptibility, and mixed-response characterization into selection algorithms and trial endpoints.
**CNA versus pacemaker in the 40–60-year-old patient**	This is the key clinical equipoise: pacing is established, whereas CNA offers a mechanism-directed, device-sparing alternative.	Perform randomized head-to-head trials comparing CNA with contemporary pacing strategies in patients who otherwise meet pacing criteria.
**Phenotype-guided versus extensive denervation**	Extensive biatrial ablation may increase lesion burden and excessive denervation, whereas overly limited ablation may leave residual nodal vagal input.	Define whether tailored target selection according to sinus-node, AV-node, or mixed phenotype provides durable efficacy with less ablation.
**Standardized procedural endpoints**	CNA lacks a binary endpoint equivalent to pulmonary vein isolation or accessory pathway block.	Validate outcome-linked, phenotype-specific endpoints, including ECVS response, atropine response, heart-rate acceleration, PR shortening, and Wenckebach-point normalization.
**Multimodal GP localization: anatomy, electrograms, imaging, and function**	No single method reliably identifies clinically relevant autonomic targets in all patients.	Determine whether convergence of anatomical landmarks, fEGM, HFS, spectral/OTNF analysis, CT-derived fat pads, ICE, and functional testing improve clinical outcomes.
**Durability, reinnervation, and late recurrence**	Reinnervation may be partial, clinically silent, or associated with late recurrence; its biological and clinical significance remains unclear.	Use long-term follow-up with serial rhythm, autonomic, and clinical assessment to link reinnervation markers with recurrent syncope.
**Long-term autonomic safety after vagal denervation**	Vagal tone may have protective cardiovascular effects; excessive denervation could produce IST, persistent tachycardia, HRV suppression, or arrhythmic vulnerability.	Systematically capture resting heart rate, HRV, IST symptoms, atrial arrhythmias, QT changes, exercise response, and late adverse events.
**Real-world implementation and patient-centered outcomes**	Outcomes in broader practice may be less favorable than expert-center series, and success should not be defined only by syncope recurrence.	Develop prospective registries with standardized training, procedural reporting, complications, syncope burden, traumatic events, quality of life, occupational impact, and cost-effectiveness.

CNA, cardioneuroablation; CT, computed tomography; ECVS, extracardiac vagal stimulation; fEGM, fractionated electrogram; GP, ganglionated plexus; HFS, high-frequency stimulation; HRV, heart-rate variability; HUTT, head-up tilt testing; ICE, intracardiac echocardiography; ILR, implantable loop recorder; IST, inappropriate sinus tachycardia; OTNF, omnipolar technology near-field.

## Data Availability

No new data were created or analyzed in this study. Data sharing is not applicable to this article.

## Abbreviations

ADAS 3D, Anatomical Detection and Ablation System 3D; AF, atrial fibrillation; AF-Nest, atrial fibrillation nest; APHRS, Asia Pacific Heart Rhythm Society; Ao, aorta; Ao-SVC GP, aorta–superior vena cava ganglionated plexus; AUC, area under the receiver-operating-characteristic curve; AV, atrioventricular; AVNRT, atrioventricular nodal reentrant tachycardia; CI, confidence interval; CLS, closed-loop stimulation; CNA, cardioneuroablation; CS, coronary sinus; CSS, carotid sinus syndrome; CT, computed tomography; DC, deceleration capacity; EQ-5D, EuroQol 5-Dimension; EQ-VAS, EuroQol visual analogue scale; ECG, electrocardiogram; ECVS, extracardiac vagal stimulation; EGM, electrogram; EHRA, European Heart Rhythm Association; fEGM, fractionated electrogram; GP, ganglionated plexus; HCN4, hyperpolarization-activated cyclic nucleotide-gated channel 4; HFS, high-frequency stimulation; HR, heart rate; HRS, Heart Rhythm Society; HRV, heart-rate variability; HU, Hounsfield unit; HUTT, head-up tilt testing; ICE, intracardiac echocardiography; ILR, implantable loop recorder; IPSGP, inferior paraseptal ganglionated plexus; IST, inappropriate sinus tachycardia; IVC, inferior vena cava; LA, left atrium/left atrial; LAA, left atrial appendage; LAHRS, Latin American Heart Rhythm Society; LIGP, left inferior ganglionated plexus; LSGP, left superior ganglionated plexus; MTGP, Marshall tract ganglionated plexus; OTNF, omnipolar technology near field; PFA, pulsed-field ablation; PMLGP, posteromedial left ganglionated plexus; PRISMA, Preferred Reporting Items for Systematic Reviews and Meta-Analyses; PV, pulmonary vein; PVI, pulmonary vein isolation; QTc, corrected QT interval; RA, right atrium/right atrial; RCT, randomized controlled trial; RF, radiofrequency; RIGP, right inferior ganglionated plexus; RI-FP, right inferior fat pad; RS-FP, right superior fat pad; RSGP, right superior ganglionated plexus; SA, sinoatrial; SF-36, 36-Item Short Form Health Survey; SND, sinus node dysfunction; SPSGP, superior paraseptal ganglionated plexus; SVC, superior vena cava; VAFIT, vagal AF induction test; VASIS, Vasovagal Syncope International Study; VVS, vasovagal syncope.

## References

[1] Serletis A., Rose S., Sheldon A.G., Sheldon R.S. (2006). Vasovagal syncope in medical and social context: A population-based study. Eur. Heart J..

[2] Vandenberk B., Lei L.Y., Ballantyne B., Vickers D., Liang Z., Sheldon R.S., Chew D.S., Aksu T., Raj S.R., Morillo C.A. (2022). Cardioneuroablation for vasovagal syncope: A systematic review and meta-analysis. Heart Rhythm.

[3] Aksu T., Brignole M., Calo L., Debruyne P., Di Biase L., Deharo J.C., Fanciulli A., Fedorowski A., Kulakowski P., Morillo C. (2024). Cardioneuroablation for the treatment of reflex syncope and functional bradyarrhythmias: A Scientific Statement of the EHRA of the ESC, the HRS, the APHRS and the LAHRS. Europace.

[4] Benabou L., Ascione C., Soré B., Cherbi M., Labrousse R., Tixier R., Bouyer B., Arnaud M., Buliard S., Pambrun T. (2025). A computed tomography-based evaluation and comparison of ganglionated plexus targeting techniques for cardioneuroablation. Heart Rhythm.

[5] Glikson M., Nielsen J.C., Kronborg M.B., Michowitz Y., Auricchio A., Barbash I.M., Barrabés J.A., Boriani G., Braunschweig F., Brignole M. (2021). 2021 ESC Guidelines on cardiac pacing and cardiac resynchronization therapy. Eur. Heart J..

[6] Pachon J.C., Pachon E.I., Pachon J.C., Lobo T.J., Pachon M.Z., Vargas R.N., Jatene A.D. (2005). “Cardioneuroablation”—New treatment for neurocardiogenic syncope, functional AV block and sinus dysfunction using catheter RF-ablation. Europace.

[7] Pachon J.C., Pachon E.I., Cunha Pachon M.Z., Lobo T.J., Pachon J.C., Santillana T.G. (2011). Catheter ablation of severe neurally mediated reflex (neurocardiogenic or vasovagal) syncope: Cardioneuroablation long-term results. Europace.

[8] Piotrowski R., Baran J., Sikorska A., Krynski T., Kulakowski P. (2023). Cardioneuroablation for Reflex Syncope: Efficacy and Effects on Autonomic Cardiac Regulation—A Prospective Randomized Trial. JACC Clin. Electrophysiol..

[9] Armani Prata A., Katsuyama E., Scardini P., Antunes V., Granja J., Coan A.C., Fukunaga C., Pachón Mateos J.C. (2025). Cardioneuroablation in patients with vasovagal syncope: An updated systematic review and meta-analysis. Heart Rhythm.

[10] Barrio-Lopez M.T., Álvarez-Ortega C., Minguito-Carazo C., Franco E., García-Granja P.E., Alcalde-Rodríguez Ó., Salvador-Montañés Ó., Francisco-Pascual J., Macías-Ruíz R., Marco Del Castillo Á. (2024). Predictors of Clinical Success of Cardioneuroablation in Patients with Syncope: Results of a Multicenter Study. JACC Clin. Electrophysiol..

[11] Tung R., Pujol-Lopez M., Locke A.H., Alyesh D.M., Sundaram S., Shah A.D., Kumar V., Kowlgi G., Kumar K., Shvilkin A. (2025). Cardioneural Ablation for Functional Bradycardia and Vasovagal Syncope: Outcomes from the U.S. Multicenter CNA Registry. JACC Clin. Electrophysiol..

[12] Chung W.H., Do D., Erbay M.I., Ajijola O.A. (2026). Cardioneural ablation: Toward achieving uniformity in nomenclature, procedural approaches, and outcome measures. Heart Rhythm.

[13] Brignole M., Aksu T., Calò L., Debruyne P., Deharo J.C., Fanciulli A., Fedorowski A., Kulakowski P., Morillo C., Moya A. (2023). Clinical controversy: Methodology and indications of cardioneuroablation for reflex syncope. Europace.

[14] Armour J.A. (2008). Potential clinical relevance of the “little brain” on the mammalian heart. Exp. Physiol..

[15] Armour J.A., Murphy D.A., Yuan B.X., Macdonald S., Hopkins D.A. (1997). Gross and microscopic anatomy of the human intrinsic cardiac nervous system. Anat. Rec..

[16] Piotrowski R., Baran J., Sikorska A., Niedzwiedz M., Krynski T., Kulakowski P. (2024). Cardioneuroablation: Comparison of acute effects of the right vs. left atrial approach in patients with reflex syncope: The ROMAN2 study. Europace.

[17] Gigante C., Penela D., Viveros D., Falasconi G., Teresi L., Latini A.C., Soto-Iglesias D., Franco-Ocaña P., Francia P., Alderete J. (2026). A tailored approach to cardioneuroablation for reflex syncope and functional bradycardia: Results from the ELEGANCE multicentre study. Europace.

[18] Tu B., Wu L., Hu F., Fan S., Liu S., Liu L., Ding L., Zheng L., Yao Y. (2022). Cardiac deceleration capacity as an indicator for cardioneuroablation in patients with refractory vasovagal syncope. Heart Rhythm.

[19] Futyma P., Zarębski Ł., Wrzos A., Futyma M., Kułakowski P. (2024). Cardioneuroablation of Right Anterior Ganglionated Plexus for Treatment of Vagally Mediated Paroxysmal Atrial Fibrillation. JACC Clin. Electrophysiol..

[20] van Dijk J.G., Sheldon R., Sutton R. (2024). Making certain that noninvasive therapy for vasovagal syncope has failed before proceeding to invasive interventions. Europace.

[21] Chung W.H., Masuyama K., Challita R., Hayase J., Mori S., Cha S., Bradfield J.S., Ardell J.L., Shivkumar K., Ajijola O.A. (2023). Ischemia-induced ventricular proarrhythmia and cardiovascular autonomic dysreflexia after cardioneuroablation. Heart Rhythm.

[22] Francia P., Viveros D., Falasconi G., Penela D., Soto-Iglesias D., Martí-Almor J., Alderete J., Saglietto A., Bellido A.F., Franco-Ocaña P. (2023). Clinical impact of aging on outcomes of cardioneuroablation for reflex syncope or functional bradycardia: Results from the ELEGANCE multicenter study. Heart Rhythm.

[23] Kulakowski P., Sikorska A., Krynski T., Niedzwiedz M., Soszynska M., Baran J., Piotrowski R. (2026). Older age is not an independent predictor of syncope recurrence following cardioneuroablation for reflex syncope. J. Interv. Card. Electrophysiol..

[24] Minguito-Carazo C., Martínez-Alday J.D., Martínez-Sande J.L., García Seara J., Fernández López X.A., Shangutov O., Larrabide Eguren I., González-Ferrero T., Elices-Teja J., Pérez Veloso M.A. (2024). Effect of age on clinical impact and mid-term denervation in patients undergoing cardioneuroablation. Sci. Rep..

[25] Lampert R., Chung E.H., Ackerman M.J., Arroyo A.R., Darden D., Deo R., Dolan J., Etheridge S.P., Gray B.R., Harmon K.G. (2024). HRS Expert Consensus Statement on Arrhythmias in the Athlete. Heart Rhythm.

[26] Choi N.H., Hong J., Moak J.P. (2024). Cardioneuroablation for pediatric patients with functional sinus node dysfunction and paroxysmal atrioventricular block. J. Cardiovasc Electrophysiol..

[27] Chipa Ccasani F., Cruzalegui J., Martinez-Barrios E., Cesar-Diaz S., Greco A.N., Merchan E.R., Cerralbo P., Diez-Escute N., Zschaeck I., Brugada J. (2025). Cardioneuroablation: An alternative to pacing in pediatric patients with bradycardia and syncope. Europace.

[28] Aksu T., Piotrowski R., Tung R., De Potter T., Markman T.M., du Fay de Lavallaz J., Rekvava R., Alyesh D., Joza J.E., Badertscher P. (2024). Procedural and Intermediate-term Results of the Electroanatomical-guided Cardioneuroablation for the Treatment of Supra-Hisian Second- or Advanced-degree Atrioventricular Block: The PIRECNA multicentre registry. Europace.

[29] Skeete J., Gordon J.S., Kavinksy L., Huang H.D., Aksu T. (2025). Cardioneuroablation for the management of neurally mediated syncope, sinus bradycardia, and atrioventricular block. J. Interv. Card. Electrophysiol..

[30] San Antonio R., Di Marco A., Mercé J., Rodríguez-García J., Rodríguez M., Faga V., Dallaglio P.D., Anguera I. (2025). Cardioneuroablation as a Therapeutic Approach for Functional AV Block Presenting Late After AVNRT Ablation. J. Cardiovasc Electrophysiol..

[31] Al-Othman S., Alturki A., Proietti R., Baranchuk A. (2024). Bradycardia in athletes: Exercise-induced or intrinsic nodal disease?. Heart Rhythm.

[32] Francia P., Viveros D., Gigante C., Falasconi G., Penela D., Soto-Iglesias D., Landra F., Teresi L., Marti-Almor J., Alderete J. (2025). Differential and synergistic effects of right and left atrial ganglionated plexi ablation in patients undergoing cardioneuroablation: Results from the ELEGANCE multicenter study. J. Interv. Card. Electrophysiol..

[33] Aksu T., Skeete J.R., Huang H.H. (2023). Ganglionic Plexus Ablation: A Step-by-step Guide for Electrophysiologists and Review of Modalities for Neuromodulation for the Management of Atrial Fibrillation. Arrhythm. Electrophysiol. Rev..

[34] Tu B., Chen A., Cai S., Zhang Z., Zhou L., Lai Z., Maimaitijiang P., Hu Z., Wu L., Ding L. (2025). The Efficacy of Left Atrial vs Biatrial Cardioneuroablation in Patients with Vasovagal Syncope: A Randomized Clinical Trial. JACC Clin. Electrophysiol..

[35] Aksu T., Tung R., De Potter T., Markman T.M., Santangeli P., du Fay de Lavallaz J., Winterfield J.R., Baykaner T., Alyesh D., Joza J.E. (2025). Cardioneuroablation for the management of patients with recurrent vasovagal syncope and symptomatic bradyarrhythmias: The CNA-FWRD Registry. J. Interv. Card. Electrophysiol..

[36] Aksu T., Po S.S. (2024). How to perform cardioneuroablation for vasovagal syncope and functional bradycardia. Heart Rhythm.

[37] Li C., Han W., Zhang L., Liu S., Li Z. (2025). High-frequency stimulation for ganglionated plexus localization during cardioneuroablation: A systematic review. BMC Cardiovasc Disord..

[38] Aksu T., Guler T.E., Bozyel S., Golcuk S.E., Yalin K., Lakkireddy D., Gopinathannair R. (2021). Medium-term results of cardioneuroablation for clinical bradyarrhythmias and vasovagal syncope: Effects on QT interval and heart rate. J. Interv. Card. Electrophysiol..

[39] Chakraborty P., Chen P.S., Gollob M.H., Olshansky B., Po S.S. (2024). Potential consequences of cardioneuroablation for vasovagal syncope: A call for appropriately designed, sham-controlled clinical trials. Heart Rhythm.

[40] John L.A., Mullis A., Payne J., Tung R., Aksu T., Winterfield J.R. (2021). Fractionation Mapping of the Ganglionated Plexi for Cardioneuroablation. J. Innov. Card. Rhythm Manag..

[41] Giomi A., Bernardini A., Paoletti Perini A., Zaccaria C.S., Padeletti M., Milli M. (2024). Cardioneuroablation guided by real-time spectral analysis: The Omnipolar Technology Near Field. Hear. Case Rep..

[42] Onder S.E., Guler T.E., Bozyel S., Cagdas M., Celik A.I., Dalgic S.N., Sipal A., Duman A.B., Kılıc E., Huang H.D. (2026). Enhanced mapping and targeting of ganglionated plexi using peak frequency analysis in cardioneuroablation. J. Interv. Card. Electrophysiol..

[43] San Antonio Dharandas R., Rodríguez Silva J., Prieto Gomà M., Garrido Vila L., Rodríguez García J., Rodríguez García M., Mercé Klein J., Almonte I., Sanaú Martín J., Mosquea R. Cardioneuroablación guiada por un enfoque multimodal para una localización más precisa de las zonas diana [Multimodal-guided cardioneuroablation for more precise target-zone identification]. Proceedings of the SEC 2025—El Congreso de La Salud Cardiovascular.

[44] Francia P., Viveros D., Falasconi G., Soto-Iglesias D., Fernández-Armenta J., Penela D., Berruezo A. (2023). Computed tomography-based identification of ganglionated plexi to guide cardioneuroablation for vasovagal syncope. Europace.

[45] Farwati M., Hussein A.A., Giraldo S., Bautista W., Higuchi K., Baranowski B., Bhargava M., Callahan T.D., Chung M.K., Chung R. (2025). Intracardiac echocardiography guided anatomical approach to cardioneuroablation: Feasibility and outcomes. Europace.

[46] Valenti N., Di Monaco A., Romanazzi I., Vitulano N., Troisi F., Quadrini F., Vitullo A., Sgarra L., Caruso R., Anzelmo V. (2025). Cardioneuroablation for reflex syncope or functional bradyarrhytmias: New insight from a single center experience. Front. Cardiovasc. Med..

[47] Falasconi G., Penela D., Soto-Iglesias D., Francia P., Teres C., Saglietto A., Jauregui B., Viveros D., Bellido A., Alderete J. (2023). Personalized pulmonary vein antrum isolation guided by left atrial wall thickness for persistent atrial fibrillation. Europace.

[48] Linz D., Stavrakis S. (2025). Cardioneuroablation for vasovagal syncope: How to move beyond “learning by burning”?. J. Interv. Card. Electrophysiol..

[49] Pachon-M E.I., Pachon-Mateos J.C., Higuti C., Santillana-P T.G., Lobo T., Pachon C., Pachon-Mateos J., Zerpa J., Ortencio F., Amarante R.C. (2020). Relation of Fractionated Atrial Potentials with the Vagal Innervation Evaluated by Extracardiac Vagal Stimulation During Cardioneuroablation. Circ. Arrhythm. Electrophysiol..

[50] Aksu T., Guler T.E., Bozyel S., Yalin K., Gopinathannair R., Tanboga I.H. (2020). Postprocedural heart rate as a predictor of clinical success in cardioneuroablation. Europace.

[51] Kulakowski P., Baran J., Sikorska A., Krynski T., Niedzwiedz M., Soszynska M., Piotrowski R. (2024). Cardioneuroablation for reflex asystolic syncope: Mid-term safety, efficacy, and patient’s acceptance. Heart Rhythm.

[52] Ababei A., Ursu C.G., Bistriceanu M.I.A., Andreescu D.I., Iurea I.M., Budeanu B., Dumitrache A.E., Hostiuc A., Sturz-Lazar M.C., Toma C.V. (2025). Cardioneuroablation for Vasovagal Syncope: An Updated Systematic Review and Single-Arm Meta-Analysis. Biomedicines.

[53] Chen A., Zhang T., Sun H., Tu B., Maimaitijiang P., Wang X., Zheng L. (2026). Efficacy and safety of cardioneuroablation as a treatment for patients with functional bradyarrhythmia: A systematic review and meta-analysis. Int. J. Cardiol. Heart Vasc..

[54] Upadhyay G.A. (2024). Predicting Clinical Success After Cardioneural Ablation for Syncope: Time to Get into the Weeds. JACC Clin. Electrophysiol..

[55] Gopinathannair R., Olshansky B., Turagam M.K., Gautam S., Futyma P., Akella K., Tanboga H.I., Bozyel S., Yalin K., Padmanabhan D. (2025). Permanent pacing versus cardioneuroablation for cardioinhibitory vasovagal syncope. J. Interv. Card. Electrophysiol..

[56] Baron-Esquivias G., Morillo C.A., Moya-Mitjans A., Martínez-Alday J., Ruiz-Granell R., Lacunza-Ruiz J., Garcia-Civera R., Gutierrez-Carretero E., Romero-Garrido R. (2017). Dual-Chamber Pacing with Closed Loop Stimulaton in Recurrent Reflex Vasovagal Syncope: The SPAIN study. J. Am. Coll. Cardiol..

[57] Brignole M., Russo V., Arabia F., Oliveira M., Pedrote A., Aerts A., Rapacciuolo A., Boveda S., Deharo J.C., Maglia G. (2021). Cardiac pacing in severe recurrent réflex syncope and tilt-induced asystole. Eur. Heart J..

[58] San Antonio R. (2025). Time for a paradigm shift: From pacing to ablation in reflex syncope. J. Interv. Card. Electrophysiol..

[59] Skoczyński P., Stec S., Ratajska A., Zając M., Hrymniak B., Kustroń A., Andrejków A., Stodółkiewicz-Nowarska E., Śledź J., Jagielski D. (2025). Cardioneuroablation in the Management of Vagally Mediated Bradyarrhythmias—A Comprehensive Review of Ongoing Randomized Controlled Trials. J. Clin. Med..

[60] Skoczyński P., Hrymniak B., Skonieczny B., Biel B., Josiak K., Krakowiak B., Ptaszkowski K., Gołba K.S., Bednarek J., Kosior J. (2024). GENTLE-PACE—A multicenter, randomized, double-blinded research study comparing the Efficacy and safety of cardioNeuroablaTion vs. permanent pacing in patients with an implantabLE PACEmaker for symptomatic bradycardia. Cardiol. J..

[61] Wichterle D., Kulakowski P. (2024). To the Editor—Cardioneuroablation: Excessive risk or excessive fear?. Heart Rhythm.

[62] Connolly S.J., Sheldon R., Thorpe K.E., Roberts R.S., Ellenbogen K.A., Wilkoff B.L., Morillo C., Gent M. (2003). VPS II Investigators. Pacemaker therapy for prevention of syncope in patients with recurrent severe vasovagal syncope: Second Vasovagal Pacemaker Study (VPS II): A randomized trial. JAMA.

[63] Raviele A., Giada F., Menozzi C., Speca G., Orazi S., Gasparini G., Sutton R., Brignole M. (2004). Vasovagal Syncope and Pacing Trial Investigators. A randomized, double-blind, placebo-controlled study of permanent cardiac pacing for the treatment of recurrent tilt-induced vasovagal syncope. The vasovagal syncope and pacing trial (SYNPACE). Eur. Heart J..

[64] Sud S., Massel D., Klein G.J., Leong-Sit P., Yee R., Skanes A.C., Gula L.J., Krahn A.D. (2007). The expectation effect and cardiac pacing for refractory vasovagal syncope. Am. J. Med..

[65] Penela D., Berruezo A., Roten L., Futyma P., Richter S., Falasconi G., Providencia R., Chun J., on behalf of EHRA Scientific Initiatives Committee (2024). Cardioneuroablation for vasovagal syncope: Insights on patients’ selection, centre settings, procedural workflow and endpoints—Results from an European Heart Rhythm Association survey. Europace.

[66] Zafeiropoulos S., Stavrakis S. (2023). Potential proarrhythmic effects of cardioneuroablation: Primum non nocere. Heart Rhythm.

[67] Kulakowski P., Piotrowski R. (2025). Inappropriate Sinus Tachycardia Following Cardioneuroablation for Reflex Syncope: A Case Report and Review of the Literature. Arrhythm. Electrophysiol. Rev..

[68] Stec S., Kuteszko R., Sulik A., Kornaszewska M. (2025). Double trouble: Hybrid management of inappropriate sinus tachycardia and sinus bradycardia in the era of cardioneuromodulation. Hear. Case Rep..

[69] Pachon-M J.C., Pachon-M E.I., Pachon C.T.C., Santillana-P T.G., Lobo T.J., Pachon-M J.C., Zerpa-A J.C., Cunha-P M.Z., Higuti C., Ortencio F.A. (2020). Long-Term Evaluation of the Vagal Denervation by Cardioneuroablation Using Holter and Heart Rate Variability. Circ. Arrhythm. Electrophysiol..

[70] Futyma P., Kułakowski P. (2022). Reinnervation after cardioneuroablation: When on the run for best intraprocedural endpoints, be aware of possible ablation overdose. Hear. Case Rep..

[71] Baysal E., Mutluer F.O., Dagsali A.E., Kumrulu U.C., Huang H.D., Aksu T. (2025). Improved health-related quality of life after cardioneuroablation in patients with vasovagal syncope. J. Interv. Card. Electrophysiol..

[72] Maimaitijiang P., Tu B., Lai Z., Chen A., Zhang Z., Zhou L., Zheng L., Yao Y. (2025). CAMPAIGN Investigators. The Efficacy of Cardioneuroablation versus Midodrine in Patients with Vasovagal Syncope: Design and Rationale for the CAMPAIGN Trial. J. Interv. Card. Electrophysiol..

[73] Stec S., Jankowska-Polańska B., Jagielski D., Wileczek A., Josiak K., Śledź J., Reichert A., Kustroń A., Zyśko D., Skonieczny B. (2022). Rationale and design of SAN.OK randomized clinical trial and registry: Comparison of the effects of evidence-based pacemaker therapy and cardioneuroablation in sinus node dysfunction. Cardiol. J..

[74] Stodółkiewicz-Nowarska E., Stec S., Wileczek A., Skoczyński P., Gościńska-Bis K., Magielski P., Zając M., Ratajska A., Kustroń A., Śledź J. (2024). TELE-SPACER—A randomized clinical trial protocol: Cardioneuroablation versus pacemaker implantation in the treatment of vagally-mediated atrio-ventricular block. Cardiol. J..

[75] Wang H., Zhang Y., Liao S., Zhang H., Huang X. (2026). Comparing Physical, Pharmacological, Pacemaker, and Cardioneuroablation Therapies for Patients with Vasovagal Syncope: A systematic review and network meta-analysis. Int. J. Cardiol. Heart Vasc..

[76] Cacciapuoti F., Crispo S., Ambrosino S., Pirozzi C., Munciguerra O., Nappo C., Caccavale N., Casolaro F., Volpicelli M. (2026). Biatrial cardioneuroablation guided by robotic magnetic navigation and artificial intelligence-based mapping in vagal bradyarrhythmias: A controlled observational study. J. Interv. Card. Electrophysiol..

[77] Pachon-M J.C., Pachon-M E.I., Santillana-P T.G., Lobo T.J., Pachon C.T.C., Pachon-M J.C., Pachon M.Z.C., Clark J. (2025). Vagal AF induction test (VAFIT): A new endpoint for optimizing atrial fibrillation ablation through cardioneuroablation. J. Interv. Card. Electrophysiol..

[78] O’Brien B., Reilly J., Coffey K., González-Suárez A., Quinlan L., van Zyl M. (2023). Cardioneuroablation Using Epicardial Pulsed Field Ablation for the Treatment of Atrial Fibrillation. J. Cardiovasc Dev. Dis..

